# Phenotypic selection with an intrabody library reveals an anti-apoptotic function of PKM2 requiring Mitofusin-1

**DOI:** 10.1371/journal.pbio.2004413

**Published:** 2019-06-10

**Authors:** Tong Liu, Tomomi Kuwana, Hongkai Zhang, Matthew G. Vander Heiden, Richard A. Lerner, Donald D. Newmeyer

**Affiliations:** 1 La Jolla Institute for Allergy and Immunology, La Jolla, California, United States of America; 2 Department of Molecular Biology, The Scripps Research Institute, La Jolla, California, United States of America; 3 David H. Koch Institute for Integrative Cancer Research and Department of Biology, Massachusetts Institute of Technology, Cambridge, Massachusetts, United States of America; University of Cambridge, United Kingdom of Great Britain and Northern Ireland

## Abstract

Bcl-2 family proteins control a decisive apoptotic event: mitochondrial outer membrane permeabilization (MOMP). To discover MOMP-regulating proteins, we expressed a library of intracellular single-chain variable fragments (scFvs) (“intrabodies”) and selected for those rescuing cells from apoptosis induced by BimS (the short isoform of Bim). One anti-apoptotic intrabody, intrabody 5 (IB5), recognized pyruvate kinase M2 (PKM2), which is expressed in cancer cells. PKM2 deletion ablated this clonogenic rescue; thus, IB5 activated a latent cytoprotective function of PKM2. This resulted not from pyruvate kinase activity per se but rather from the formation of an active tetrameric conformation of PKM2. A stably tetrameric PKM2 mutant, K422R, promoted cell survival even in the absence of IB5, and IB5 further increased survival. Mitochondria isolated from IB5-expressing cells were relatively resistant to MOMP in vitro. In cells, IB5 expression up-regulated Mitofusin-1 (Mfn1) and increased mitochondrial length. Importantly, Mfn1 deficiency abrogated IB5’s cytoprotective effect. PKM2’s anti-apoptotic function could help explain its preferential expression in human cancer.

## Author summary

Proteins belonging to the Bcl-2 family regulate a common form of cell death known as apoptosis. Typically, these proteins function in apoptosis by controlling the formation of large pores in the mitochondrial outer membrane (MOM). While many proteins that regulate apoptosis have been identified over the years, some may still be unknown. Here, we used an unbiased approach in which we first expressed in cultured tumor cells a library of intracellular single-chain antibodies termed “intrabodies.” We then selected for intrabodies that allowed cells to evade apoptosis. We identified pyruvate kinase isoform M2 (PKM2), a major glycolytic enzyme that has been linked to cancer development, as the specific target of one such anti-apoptotic intrabody. We showed that the PKM2-specific intrabody promoted cell survival not by neutralizing its target but rather by activating an anti-apoptotic function of PKM2. While this cell survival function of PKM2 was not related to changes in the levels of Bcl-2 family proteins or to effects on the enzymatic activity of PKM2, we found that cell survival requires the increased expression of a MOM protein, Mitofusin-1 (Mfn1), known to regulate mitochondrial fusion. We conclude that this cell survival function of PKM2 could contribute to a role in cancer progression for this protein.

## Introduction

Apoptosis is a cellular suicide process that is important for certain aspects of normal animal development [1] and is dysregulated in various diseases, especially cancer [e.g., 2,3]. Members of the Bcl-2 protein family act at the mitochondrial outer membrane (MOM) to regulate the central events in apoptotic cell death [4–13]. Venetoclax, a drug targeting Bcl-2, is currently approved for the treatment of a refractory form of chronic lymphocytic leukemia [14,15], and other drugs that directly target Bcl-2-family proteins are now in cancer clinical trials [16–18].

Bcl-2 family proteins function in a complex network of heterodimeric interactions that collectively decide between cell survival and death [12]. Several Bcl-2 subfamilies carry out different functions [19]. In particular, the proteins Bax and Bak comprise the effector subfamily responsible for the critical mitochondrial events in cell death. Genetic and in vitro studies [6,20–23] have shown that Bax/Bak can be activated by transient interactions with other Bcl-2 family proteins belonging to the “Bcl-2 homology domain 3 (BH3)-only” category (including Bim, Bid, Puma, and others.) Once activated, Bax/Bak undergo conformational changes to become fully integrated in the MOM. As a result, these proteins produce large lipidic membrane pores [24,25], in an event known as mitochondrial outer membrane permeabilization (MOMP) [13,19,26]. MOMP allows soluble mitochondrial proteins (e.g., cytochrome c, Smac, and Omi) to escape into the cytoplasm, where they trigger the activation of caspase proteases that carry out the cell death program. MOMP and cell death are decisively regulated by Bcl-2 family interactions [6,21,27,28], and this underlies the importance of targeting these proteins for cancer therapy. In this regard, Letai and colleagues have shown that the in vitro response of mitochondria from patient tumor samples to BH3 domain peptides can often predict the effect of therapy [29–31].

Bcl-2 family members can also be regulated by proteins outside the Bcl-2 family. For example, p53 can act at mitochondria both to activate Bax directly and to sequester Bcl-xL [32]. Similarly, the Retinoblastoma protein pRB is reported to translocate to mitochondria to promote Bax activation in a nontranscriptional manner [33], and oncogenes such as Myc and Ras also modulate the expression of key Bcl-2-family proteins [34]. The ability of proto-oncoproteins to inhibit or activate apoptosis is an important facet of their homeostatic function, inasmuch as cell death serves as a critical counterbalance to cell proliferation.

To discover molecules regulating the core mechanism of mitochondria-dependent cell death, we developed an unbiased functional selection approach that used libraries of “intrabodies”: intracellularly expressed single-chain antibodies (scFv). We found that some of the selected intrabodies specifically recognized a key metabolic regulatory protein, pyruvate kinase M2 (PKM2). This suggests that PKM2, aside from its well-documented role in glycolytic metabolism, could also have an expressly anti-apoptotic function.

PKM2 is an important regulator of tissue homeostasis as well as tumor growth and metabolism [e.g., 35] and is currently a subject of intense research [reviewed in 36,37–39]. PKM2 is a glycolytic enzyme that promotes the “Warburg effect,” also termed aerobic glycolysis, in which cells exhibit increased glucose to lactate conversion even in the presence of oxygen [40]. In cancer cells, PKM2 is typically expressed preferentially over its related isoform PKM1, even when the tissue of origin does not express PKM2. Hypothetically, cancers gain some selective advantage from the highly regulated functions of PKM2. The adaptive metabolic functions of PKM2 also come into play in some cell types that quickly transition to a proliferative state, such as lipopolysaccharide (LPS)-activated macrophages [41].

PKM1 and PKM2 are generated from transcripts of the PKM gene by alternative mRNA splicing. Both isoforms can catalyze the last step in glycolysis, in which phosphoenolpyruvate (PEP) and ADP are converted to pyruvate and ATP. Isoforms M1 and M2 are identical except for the region encoded by the one alternatively spliced exon (exon 9 for PKM1 and 10 for PKM2), yielding a difference in only 22 amino acids. PKM1 exists as a constitutively active tetramer, whereas PKM2 is subject to many forms of regulation. Various metabolites, including fructose-1,6-bisphosphate (FBP), serine, phenylalanine, and triiodo-L-thyronine (T3), can allosterically regulate PKM2’s glycolytic activity [40,42]. In vitro biochemical studies have shown that PKM2 exists in equilibrium between a glycolytically active tetramer form and less active dimer or monomer forms [43,44]. Based on crystallographic data, it has also been proposed that PKM2 tetramers can transition between inactive T-state and active R-state conformations [45].

Paradoxically, it is the ability of PKM2’s glycolytic activity to be reduced that favors rapid cell proliferation. Reduced PK activity correlates with increased biosynthesis of metabolites important for cell proliferation, potentially explaining why tumor cells prefer the M2 isoform [40,46]. Consistent with this idea, treatment of cells with small-molecule activators of PKM2 [47,48] or the replacement of PKM2 with the constitutively active isoform PKM1 [35] can reduce cell proliferation in some situations. In primary Mouse Embryonic Fibroblasts (MEFs), deletion of PKM2 results in increased PKM1 expression, and this in turn impairs nucleotide availability for DNA synthesis, thereby inhibiting cell cycle progression [49].

PKM2 is reported also to have nonglycolytic functions. Many PKM2 interaction partners have been described, including multiple transcription factors [50]. For example, PKM2 is reported to cooperate with Hif-1α to regulate the transcription of multiple glycolysis-related proteins, which contribute to metabolic remodeling and the Warburg effect [41,51–53]. These transcriptional functions require the nuclear import of PKM2 [52–55]. PKM2’s nuclear translocation can be promoted by epidermal growth factor receptor (EGFR) activation [56] and regulated by Erk1/2 and JMJD5 [57,58]. In the nucleus, PKM2 can promote β-catenin transactivation, leading to the expression of cyclin D1 and tumorigenesis [56]. A PKM2-activating compound, TEPP-46, which causes PKM2 tetramerization, inhibits Hif-1α–dependent transcriptional effects [41], supporting the idea that the dimeric form of PKM2 is responsible for transcriptional functions. Dimeric PKM2 is also reported to possess protein kinase activity, targeting multiple oncogenic factors [54,59–61]. However, PKM2 protein kinase activity is controversial, as Vander Heiden and colleagues found no evidence of protein kinase activity for PKM2 in cell lysates [62].

In some cases, PKM2 ablation can produce or enhance cell death [63–69]. Precisely how PKM2 affects apoptosis is unclear. PKM2 silencing has been reported to stabilize proapoptotic Bim [70] or down-regulate the expression of the anti-apoptotic proteins Bcl-xL or Mcl-1 [71,72]. However, PKM2 knockdown produces an artificial situation. PKM2 has multiple functions that may be regulated independently, and experiments in which this protein is ablated would involve a simultaneous loss of all of these activities, along with a compensatory up-regulation of PKM1, making interpretation difficult. In contrast to the studies just mentioned, Sabatini and colleagues showed that the inhibition of PKM2 activity under ischemic conditions had the effect of promoting cell survival rather than cell death [73]. The cells bordering necrotic foci in gliomas expressed higher levels of the enzyme SHMT2, leading to an allosteric inhibition of PKM2’s glycolytic activity. This provided a significant protection from ischemic cell death. In another ischemia model, these authors found that overexpression of PKM2 or treatment with the PKM2-activating compound TEPP-46 eliminated the increased cell viability produced by SHMT2. It is unclear whether this connection between reduced PKM2 activity and survival is a general phenomenon or only applies to certain cancer cell subsets or environments.

In contrast to studies emphasizing PKM2 loss of function, our results now show that PKM2 possesses a positive cytoprotective function that can be activated by a PKM2-specific intrabody We show that this latent function of PKM2 counteracts the central Bax/Bak-dependent mitochondrial apoptotic mechanism. Moreover, the stably tetrameric mutant PKM2 (K422R) supported intrabody 5 (IB5)’s cytoprotective effect, arguing that the anti-apoptotic function involves the cytoplasmic tetramer form rather than the nuclear dimer form of PKM2. The K422R mutant also produced BimS resistance in MEFs after expression for several passages, even in the absence of IB5. This mutant’s ability to counteract the central apoptotic pathway could provide a selective advantage for these cells, and indeed, this mutation was found to be spontaneously selected in Bloom syndrome patient tumor cells. The IB5/PKM2-induced cytoprotective function depended in part on up-regulation of the mitochondrial fusion–related protein Mitofusin-1 (Mfn1). Therefore, we propose that PKM2 can activate an Mfn1-dependent general anti-apoptotic pathway, which could help explain why human cancer cells often preferentially express the M2 isoform of PK.

## Results and discussion

Molecules regulating the core apoptotic machinery could be important in a variety of pathological and physiological situations. To discover such proteins, we adapted a functional selection approach that had been developed to identify proteins with various intracellular functions [74–76]. First, we infected HEK293T (293T) cells with a lentiviral library of genes encoding “intrabodies” [76]: intracellularly expressed scFvs. These molecules consisted of the variable regions from immunoglobulin heavy (V_H_) and light (V_L_) chains, connected by a flexible peptide linker, which formed a naïve human combinatorial scFv lentiviral library (diversity 4.5 x 10^9^). We induced apoptosis in the cells by transiently transfecting them with a cDNA encoding BimS, the most potent pro-apoptotic isoform of Bim. Bim is one of the most important BH3-only proteins and is required for cell homeostasis in numerous physiological settings. Importantly, BimS promotes cell death by acting in the central apoptotic death mechanism, both by activating Bax/Bak and by sequestering anti-apoptotic Bcl-2 family members [21,27]. Bax/Bak activation then produces MOMP, crista junction remodeling, and apoptosis [6,77].

**Fig 1:**
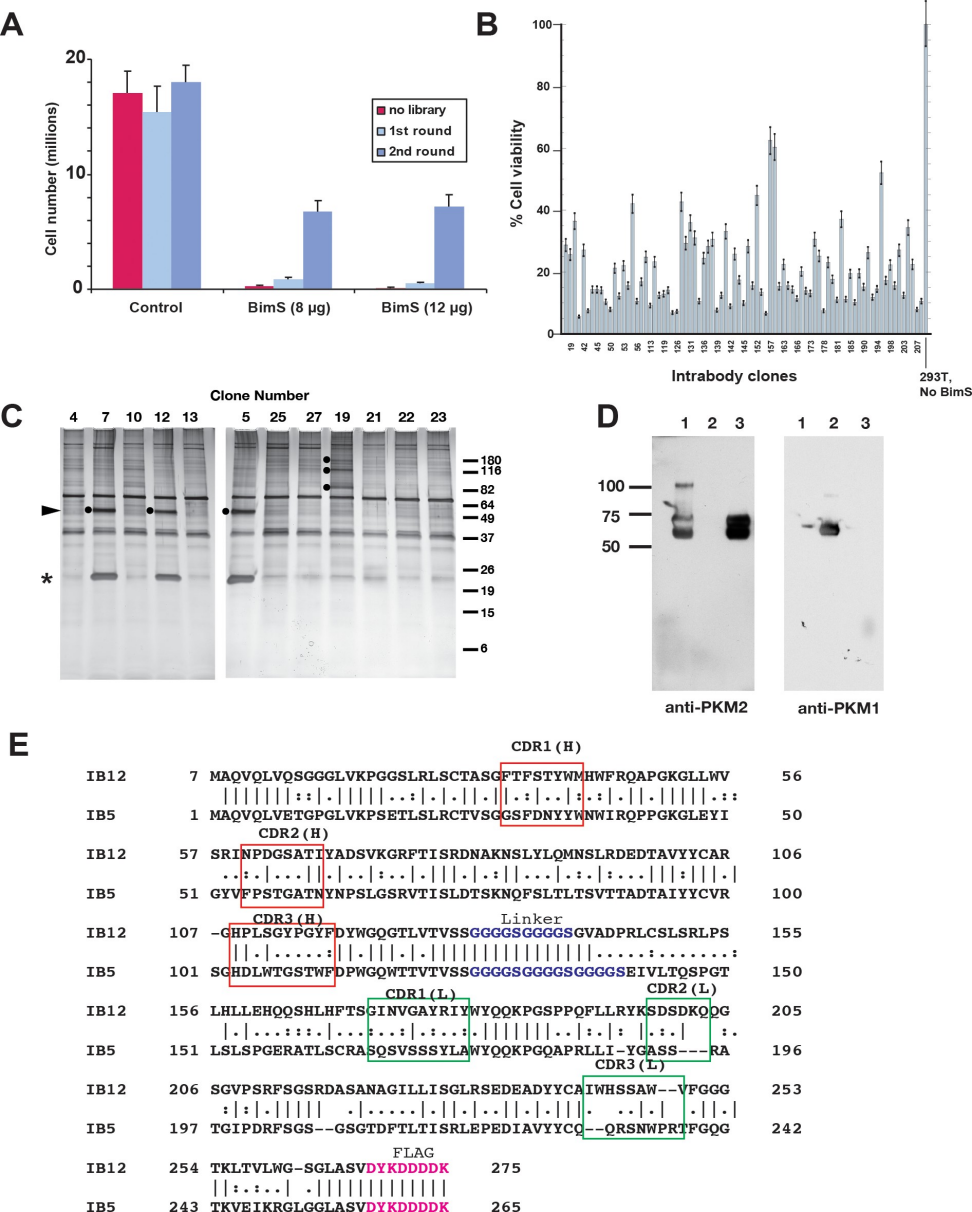
Selection of intrabodies that rescue cells from BimS-induced apoptosis identifies PKM2 as a target. A. Enrichment in two rounds of selection. For the first round, 293T cells were first infected with a lentiviral human naïve scFv library. Then, 1 x 10^5^ of the scFv-expressing cells were transiently transfected with BimS (using either 4 μg/ml or 6 μg/ml of plasmid DNA input per reaction) under the control of the EF-1α promoter, as indicated in Materials and methods. Lentiviral DNA was recovered from these rescued cells and used for a second round of selection. In the second round, many more cells (approximately 40%) were rescued from BimS-induced apoptosis. B. Individual scFv genes protected cells to various extents from BimS-induced apoptosis. After three rounds of selection, intrabody-coding sequences were amplified by PCR and subcloned into a plasmid for expression in *E*. *coli*. Individual DNA sequences were sequenced and expressed in 293T cells for testing of their ability to protect cells from apoptosis induced by transfection with BimS. The percentage of viable cells, relative to cells not transfected with BimS, was assayed. C. Some intrabodies arising from the selection procedure immunoprecipitated specific cellular proteins. Intrabodies that rescued cells from BimS-induced death were chosen for pull-down analysis, as described in Materials and methods. Left panel: Triton-X-100 (1%) cell extracts were incubated with anti-FLAG beads, then proteins were eluted with 3xFLAG peptide and separated by SDS-PAGE with silver stain. Specific bands are marked with dots; some bands (e.g., at 37 and 70 kD) are nonspecific. Clones 5, 7, and 12 (independent isolates) pulled down a 55-kD protein now identified as PKM2, while clone 19 pulled down several specific bands (not studied here). The bands near 25 kD are the scFv polypeptides, whose expression levels varied. D. IB5 recognized PKM2. Shown is an IP-western of lysates from cells expressing IB5. The lysates were incubated with anti-FLAG beads, and coprecipitating proteins were eluted with FLAG_2_ peptide (lane 3). Immunoblots were probed with antibody to PKM2 (left) or PKM1 (right). Purified PKM2 (lane 1) and PKM1 (lane 2) were controls for antibody specificity. E. Protein sequences of IB5 and IB12 are dissimilar, underscoring the functional importance of their common target, PKM2. Red boxes, heavy-chain CDRs; green boxes, light-chain CDRs; magenta type, FLAG tag. The selected intrabody plasmids were sequenced by Sanger sequencing. Sequences were analyzed with Vbase2. Note: underlying data are included in corresponding tabs in the accompanying supplemental Excel file, S1 Data. 293T, HEK293T; CDR, Complementarity Determining Region; EF-1α, elongation factor 1α; IB, intrabody; PKM1/M2, pyruvate kinase isoform M1/M2; scFv, single-chain variable fragment.

We then selected for intrabodies that rescued cells from BimS-induced death by recovering scFv-encoding DNA from the surviving cells. This selection process was efficient, as BimS transfection killed approximately 99% of the control cells, whereas expression of the lentiviral scFv library rescued a small percentage of the cells (Fig 1A). We then recovered the scFv-encoding DNA from surviving cells, with which we created a new lentiviral library for a second round of selection. In this round, intrabodies rescued approximately 40% of the cells from BimS-induced death, implying a substantial enrichment of intrabodies with prosurvival activity (Fig 1A). A third round of selection did not increase the percentage of cell survival. This was not surprising, given the likelihood that many of the recovered intrabodies would promote survival with less than 100% efficiency.

To identify intrabodies that inhibit apoptosis, we isolated single intrabody-encoding genes by subcloning the enriched DNA from the second round into bacteria. We introduced approximately 300 of these individual scFv genes separately into 293T cells. As expected, many of the intrabodies rescued 293T cells from apoptosis induced by BimS expression, to varying extents (Fig 1B). To identify protein targets, we performed FLAG pull-downs of some of the intrabody-target protein complexes, which we resolved on silver-stained SDS-polyacrylamide gels. We found that some intrabodies precipitated specific cellular proteins (Fig 1C). By matrix-assisted laser desorption/ionization-time-of-flight (MALDI-TOF) mass spectrometry and immunoblot analysis (Fig 1D), we identified one protein target of three different scFv-encoding DNA clones (5, 7, and 12) as PKM2. scFvs 5 and 7 had an identical DNA sequence, whereas scFv 12 was different (the light-chain Complementarity Determining Regions [CDRs] of scFv 12 were essentially distinct from those of scFv 5, and heavy-chain CDRs were only approximately 30% identical; Fig 1E). This apparent convergent selection underscores the potential importance of PKM2 as an apoptosis-regulating target protein. We chose IB5 for further study. The specific pull-down of PKM2 and not PKM1 suggests that intrabody binding might involve residues encoded in exon 10, which is unique to PKM2.

**Fig 2:**
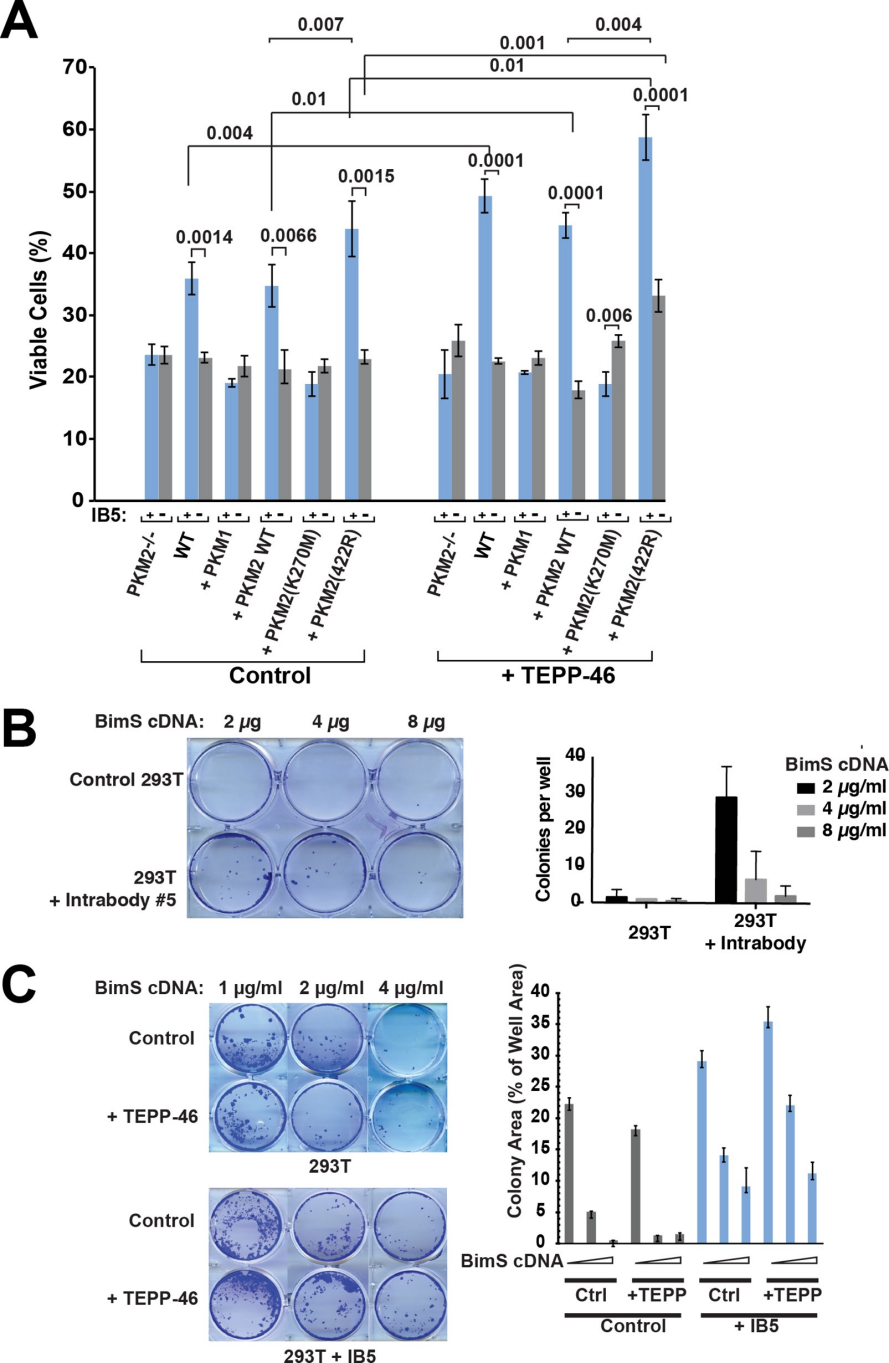
Genetic deletion of PKM2 abrogated the protective effect of scFv 5. Reconstitution of PKM2-deficient MEFs with PKM2 WT or PKM2 (K422R), but not PKM1 or PKM2 (K240M), restored cell death protection. The PKM2-activating compound TEPP-46 enhanced the clonogenic survival effect of IB5. A. Viability assays with reconstituted cells. WT MEFs, PKM2-deficient MEFs, or PKM2-deficient MEFs reconstituted with WT or mutant PKM2 cDNA were infected or not with IB5, then later transfected with BimS expression plasmid. Surviving cells were counted 48 h afterwards. Note that only WT cells or PKM2-deficient MEFs reconstituted with WT PKM2 exhibited cytoprotective activity of IB5. B. IB5 produced clonogenic survival despite BimS expression. Control or IB5-expressing cells were transfected with BimS cDNA, and after 5 d, the plates were fixed with 6.0% glutaraldehyde and stained with 0.5% crystal violet. Top: example crystal violet-stained plate; bottom: average colony counts from three independent experiments, ± SEM. C. IB5-induced clonogenic rescue of 293T cells from BimS was enhanced by treatment with TEPP-46. Control 293T or IB5-expressing cells were incubated with or without 27 g/ml TEPP-46 for 3 h, then transfected with BimS cDNA in a further 24-h incubation also including TEPP-46 or vehicle. The plates were fixed and stained with crystal violet after 1 week. Left: examples of crystal violet-stained plates; right: as cells did not typically grow as discrete colonies, we quantified the total area of colonies (a measure of the total number of proliferating cells) formed in each well using ImageJ software [78]; mean ± SEM are shown from three independent experiments. Note: underlying data are included in corresponding tabs in the accompanying supplemental Excel file, S1 Data. 293T, HEK293T; IB, intrabody; MEF, Mouse Embryonic Fibroblast; PKM1/M2, pyruvate kinase isoform M1/M2; scFv, single-chain variable fragment; WT, wild-type.

To verify that PKM2 is indeed the functional target of IB5, we first used small interfering RNA (siRNA) to silence PKM2 in 293T cells and saw a substantial reduction in the ability of IB5 to rescue cells from BimS killing (S1 Fig). Next, we expressed IB5 in MEFs genetically deficient for PKM2 [49]. Human and mouse PKM2 display approximately 98% sequence identity; thus, IB5 was expected to cross-react with the mouse protein. Indeed, IB5 rescued a percentage of the wild-type (WT) cells from BimS-induced killing, but this rescue was entirely abrogated in PKM2-deficient MEFs (Fig 2A). Moreover, reconstituting the PKM2-null MEFs with cDNA encoding WT PKM2, but not PKM1, restored the cytoprotective ability of IB5 (Fig 2A). PKM2-null MEFs are known to up-regulate expression of PKM1 [49], further confirming the specificity of the effect for PKM2. We conclude that IB5 does not neutralize PKM2 but rather acts positively to stimulate an anti-apoptotic function of PKM2. The intrabody IB5 likely mimics an unidentified physiological interaction partner of PKM2 that activates this cytoprotective function.

IB5, like a number of our intrabody hits, rescued a moderate percentage (approximately 15%–20%) of the 293T cells from BimS-induced death. We suspect that it is a tall order for intrabodies to be very potent. These molecules are monovalent and thus do not have the enhanced avidity of IgG or IgM. Also, to have an effect revealed by selection, they would likely need to be expressed as abundantly as their target proteins and therefore might be present in limiting amounts. Consistent with this possibility, when we expressed IB5 with a weaker promoter, we found that its survival effect was reduced. In any case, this cell survival is potentially significant, given how potently and directly BimS activates the central mitochondrial pathway. Importantly, we found that the PKM2-specific intrabody produced clonogenic survival, meaning that the surviving cells were able to proliferate (Fig 2B and 2C). Thus, this latent anti-apoptotic activity of PKM2 could be consequential for physiological situations, e.g., for the progression of preneoplastic cells. If even a fraction of these survive, they could ultimately undergo further adaptations, leading to oncogenesis.

**Fig 3:**
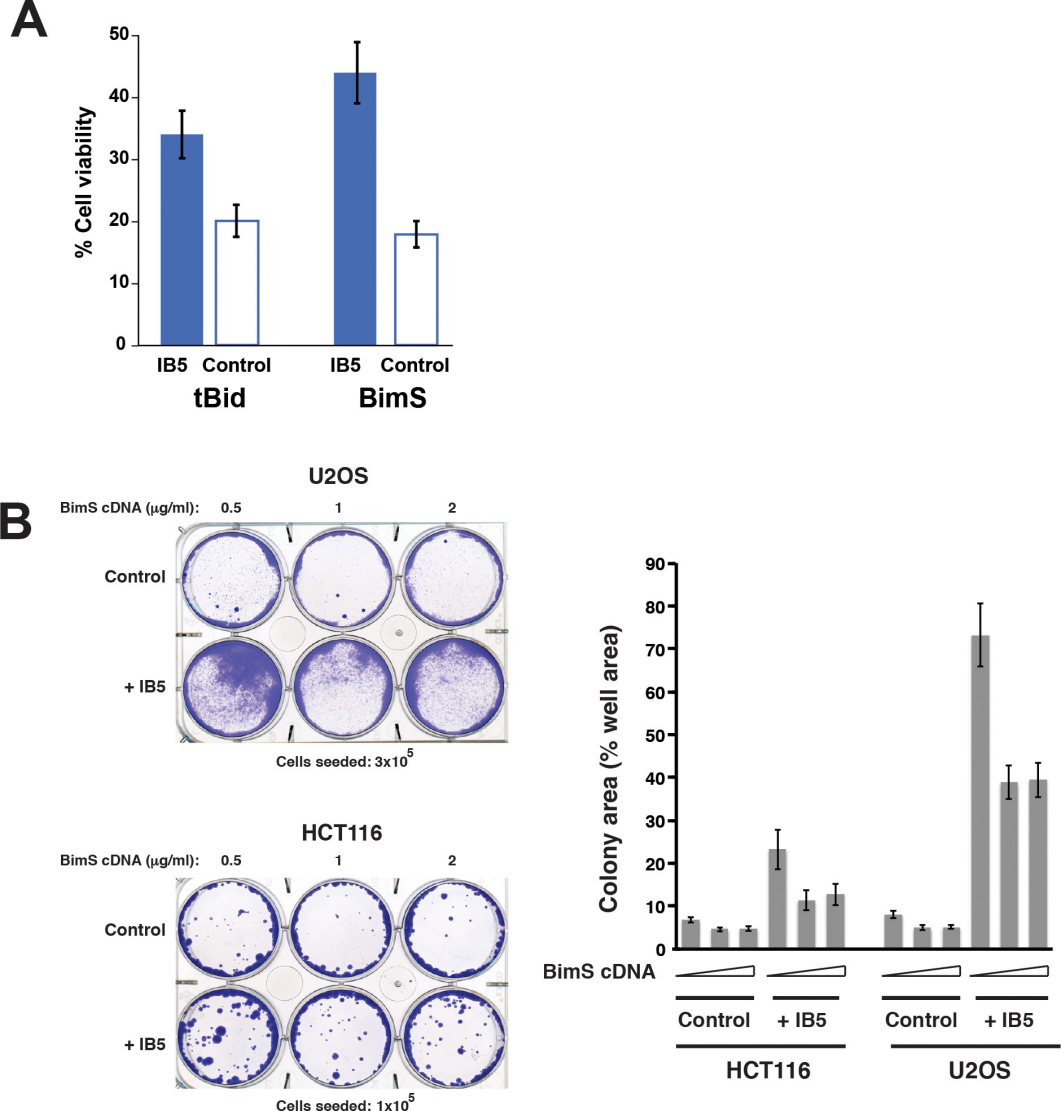
IB5 expression rescued 293T cells from death induced by transient expression of the potent pro-apoptotic proteins BimS and tBid. A. IB5-expressing 293T were protected from death induced by transient expression of tBid or BimS. 293T cells were transfected treated with 1 g/ml tBid or BimS cDNA. Surviving cells were counted after 72 h. B. IB5 expression rescued U2OS and HCT116 cells clonogenically from BimS-induced death. Left: examples of crystal violet-stained plates; right: as cells did not typically grow as discrete colonies, we measured colony area as a percentage of total plate area. Note: underlying data are included in corresponding tabs in the accompanying supplemental Excel file, S1 Data. 293T, HEK293T; IB5, intrabody 5; tBid, truncated Bid.

We found that the PKM2-specific intrabody protected cells from death induced by another potent BH3-only protein, truncated Bid (tBid; Fig 3A). As Bim and Bid are two major activators of the core apoptotic pathway, directly upstream of Bax/Bak activation and mitochondrial permeabilization, this suggests that PKM2 can inhibit the common apoptotic pathway at the level of Bax/Bak activation. If so, PKM2 could oppose physiological cell death triggered by multiple pro-apoptotic pathways. Indeed, we found that IB5 promoted clonogenic survival in cells treated with the DNA-damaging drug etoposide, although not with another cytotoxic agent, staurosporine (S2 Fig). The reasons for this are unclear, but we note that staurosporine is a promiscuous protein kinase inhibitor and presumably acts pleiotropically. Moreover, staurosporine was reported to activate both the intrinsic and the extrinsic (death receptor–mediated) pathways of apoptosis [79] and might therefore be able to bypass the requirement for Bax/Bak activation in these cells.

In further experiments, we found that IB5 substantially protected other tumor cell lines, U2OS and HCT116, from BimS-induced apoptosis (Fig 3B). However, anti-apoptotic function was cell type–specific, as IB5 failed to rescue two breast cancer–derived cell lines (parental MDA-MB231 and a lung metastatic derivative MDA-MB231-LM2) from BimS-induced cell death (S3 Fig).

We ruled out the explanation that IB5 expression could alter the intracellular levels of PKM2 (S4 Fig). We could not determine whether IB5 expression altered the expression level of the exogenous BimS cDNA because the control condition, in which IB5 was absent, produced cell death in almost all of the BimS-expressing cells. However, an effect on BimS levels is unlikely, as IB5 expression did not affect levels of endogenous Bim EL and L isoforms in normal 293T cells. Moreover, Bim EL, L, and S isoforms were detectable in the BimS-resistant cell population rescued by IB5 (S4 Fig).

### Enhanced PKM2 glycolytic activity is not the sole explanation for the anti-apoptotic effect of IB5

**Fig 4:**
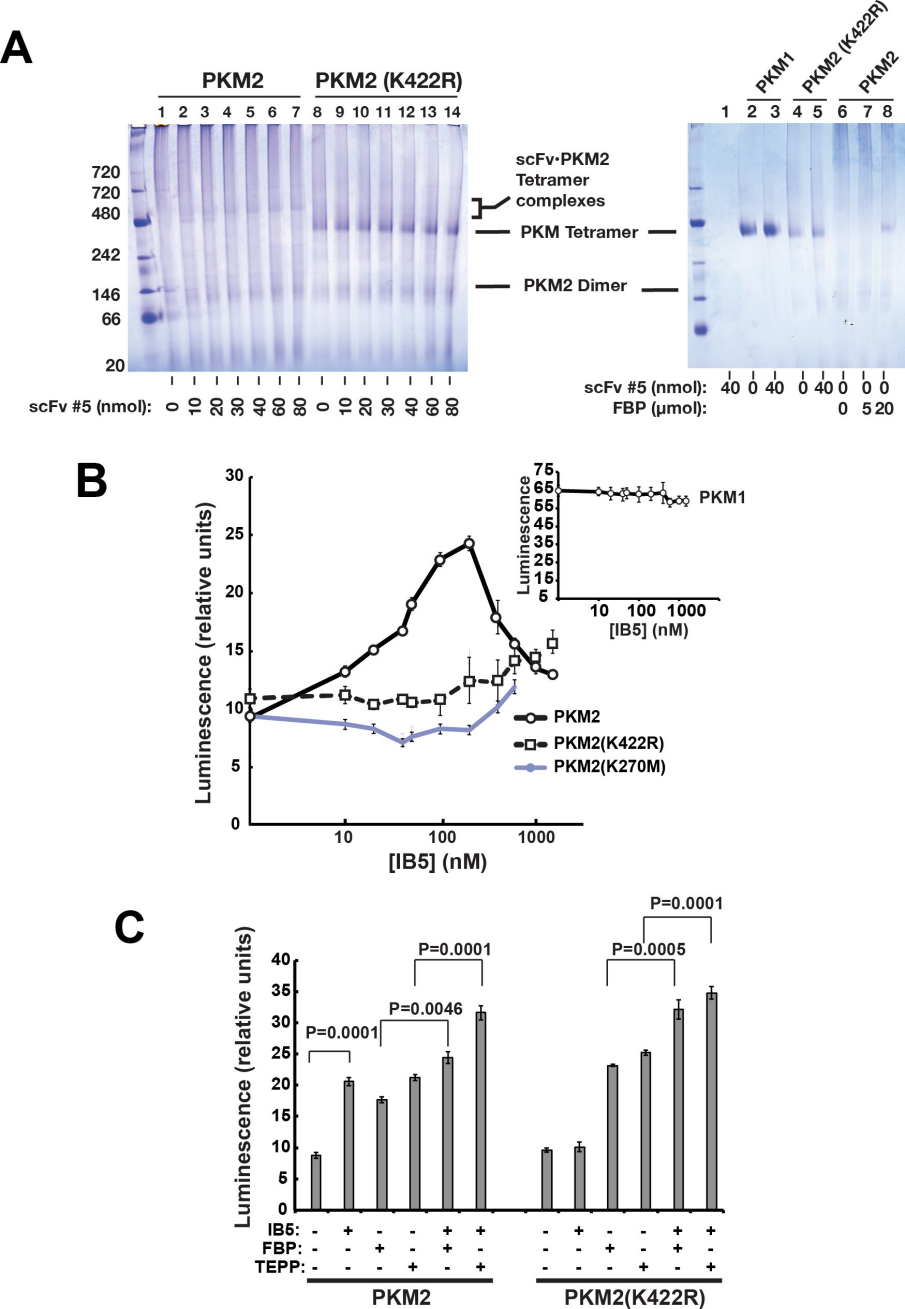
Recombinant scFv 5 induced tetramerization of recombinant PKM2 and stimulated its glycolytic activity in vitro. A. Blue native gel electrophoresis. This method resolved the dimer and tetramer forms of PKM2. Left: A monovalent form of scFv 5, produced in *E*. *coli*, induced tetramer formation in WT PKM2 along with a bandshift whose magnitude was dependent on the input amount of scFv 5. The mutant PKM2 (K422R) was constitutively tetrameric and did not exhibit a bandshift in the presence of scFv 5. Reaction volume was 20 μl. Right: scFv 5 did not produce a band shift with recombinant PKM1, which ran as a tetramer; added FBP produced the tetramer form of WT PKM2 (lanes 6–8). B. scFv 5 stimulated glycolytic activity of WT and mutant PKM2. Activity was measured by Kinase-Glo Plus Luminescent Kinase Assay kit (Promega), using ADP and PEP as substrates, with PKM2 at 50 nM. Shown are values with the basal activity of PKM2 alone subtracted out. Inset: scFv 5 did not affect PKM1’s glycolytic activity. C. IB5 stimulated the glycolytic activity of K422R mutant PKM2 to a greater extent in the presence of allosteric activators FBP or TEPP-46. Note: underlying data are included in corresponding tabs in the accompanying supplemental Excel file S1 Data. FBP, fructose-1,6-bisphosphate; IB5, intrabody 5; PEP, phosphoenolpyruvate; PKM2, pyruvate kinase isoform M2; scFv, single-chain variable fragment; WT, wild-type.

Because glucose metabolism can influence cell survival [52,80–82], we asked whether IB5 could rescue cells simply through stimulating PKM2’s glycolytic activity. First, to analyze the antibody’s interaction with PKM2 in vitro, we produced both PKM2 and a monovalent scFv corresponding to IB5 (scFv 5) as recombinant proteins in *E*. *coli*. We then mixed these proteins at various ratios and analyzed them by blue native gel electrophoresis (Fig 4A). We found that monovalent scFv 5 strongly increased the tetrameric PKM2 species and shifted up the tetramer band to a degree dependent on the molar input ratio of scFv. Thus, the antibody bound directly to PKM2, promoting its stable tetramerization. Moreover, increasing the input ratio of scFv:PKM2 gradually altered PKM2’s electrophoretic mobility, apparently reflecting the binding stoichiometry. This altered mobility in native gels might indicate either an increased mass or an altered tertiary conformation of the PKM2 tetramers.

We then found that purified scFv 5 stimulated PKM2’s glycolytic activity in a concentration-dependent manner (Fig 4B), confirming indirectly that the scFv interacts with PKM2. The activity declined at higher concentrations of scFv 5. This might result from PKM2 aggregation. These data suggest that scFv 5 activates PKM2 allosterically. (Note also that scFv 5 altered neither the electrophoretic mobility nor the PK activity of PKM1, consistent with IB5’s specificity for PKM2.)

Based on these results, we considered the possibility that the anti-apoptotic effect of IB5 purely reflected an increased glycolytic activity of PKM2. However, we found that treating cells with the PKM2-activating compound TEPP-46 (Fig 2A and 2C) [47] alone did not protect cells from BimS-induced death. Similarly, reconstituting PKM2-null MEFs with the constitutively active PKM1 failed to rescue cells (Fig 2A). Thus, high PK activity by itself was insufficient to produce an anti-apoptotic effect. On the other hand, culturing 293T cells in the presence of TEPP-46 enhanced the prosurvival effect of IB5 expression to a modest but statistically significant extent (Fig 2A and 2C). This could mean that increased glycolytic activity does contribute somewhat to cell survival. Alternatively, the effect of IB5, alone or in combination with TEPP-46, might result from stabilizing a tetrameric conformation of PKM2, whose glycolytic activity may be irrelevant to the survival function.

### Studies with PKM2 mutants

To help define the aspects of PKM2 function required for the IB5-induced anti-apoptotic effect, we reconstituted PKM2-null MEFs with WT or mutant forms of PKM2. As expected, WT PKM2 increased cell viability when coexpressed with IB5 (Fig 2A). We then analyzed the K270M mutation, reported to be catalytically dead [52,83,84]. This mutant indeed lacked basal glycolytic activity in vitro, but the addition of high concentrations of scFv 5 stimulated its PK activity somewhat (Fig 4B). PKM2 (K270M) failed to support the cytoprotective effect of IB5 (Fig 3A). If the K270M mutation merely inactivated the catalytic site, this would suggest that PKM2’s glycolytic activity is required for the cell survival effect. However, using blue native gel electrophoresis, we found that the K270M mutation also prevented the protein from forming stable tetramers in vitro, when incubated with scFv 5 and/or FBP (S5A Fig). These results have at least three possible explanations (which are not mutually exclusive): 1) PKM2’s glycolytic activity is required for the anti-apoptotic function, 2) a specific tetrameric conformation of PKM2 that produces a nonglycolytic activity is required, or 3) IB5 binding is reduced by the K270M mutation. We note that IB5 seems to have some affinity for PKM2 (K270M) because higher concentrations of scFv 5 stimulated this mutant’s glycolytic activity (Fig 4B).

We analyzed another mutant, PKM2 (K367M), which also was reported to be inactive for glycolysis [56]. We confirmed that recombinant PKM2 (K367M) indeed had little PK activity, even when incubated with scFv 5 or FBP (S5B Fig). Furthermore, like K270M, K367M lacked anti-apoptotic activity when coexpressed with IB5 (S5C Fig). However, blue native gel electrophoresis showed that recombinant K367M mutant protein did not form stable tetramers when mixed with scFv 5 (S5A Fig). Furthermore, when both scFv 5 and FBP were added, this mutant primarily formed an aberrantly migrating species, possibly a malformed tetramer. (We did also observe a minor species migrating with authentic tetramers.) Thus, because the K270M and K367M mutations impaired both PKM2’s glycolytic activity and tetramerization in vitro, the data did not resolve whether PKM2’s glycolytic activity is required for the IB5-induced cytoprotective effect.

In any case, taking together our observations that treating cells with the PKM2-activating compound TEPP-46 or replacing PKM2 with the constitutively active PKM1 failed to rescue cells from BimS-induced death, we conclude that high PK activity alone is insufficient for cell rescue by IB5. In addition, we found that the addition of 2-deoxyglucose (20 mM) to the cultures did not prevent cell rescue by IB5 (S6 Fig), suggesting that glycolysis in general was unnecessary for the cytoprotective effect of IB5.

We next considered the possibility that IB5-induced PKM2 tetramerization per se is important for the cell survival activity. To test this, we reconstituted PKM2-deficient MEFs with the stably tetrameric PKM2 (K422R) mutant. As we confirmed in Fig 4C, this mutant is glycolytically inactive, unless an allosteric activator such as FBP is added, causing a quaternary conformational change from T-state to R-state [45]. Blue native gel electrophoresis confirmed the spontaneous formation of K422R tetramers in the absence of FBP (Fig 4A). It is unclear why IB5 did not shift up the PKM2 (K422R) tetramer band, whereas it did shift up the WT tetramer. We speculate that the tertiary structure of this mutant tetramer is more rigid than that of WT PKM2. Nevertheless, it does appear that IB5 interacts with PKM2 (K422R), as the recombinant scFv stimulated this mutant’s glycolytic activity (Fig 4B), especially upon the addition of FBP or TEPP-46 (Fig 4C). It should be noted that within cells, the allosteric activator FBP is likely to be present, at concentrations dependent on the metabolic state.

**Fig 5:**
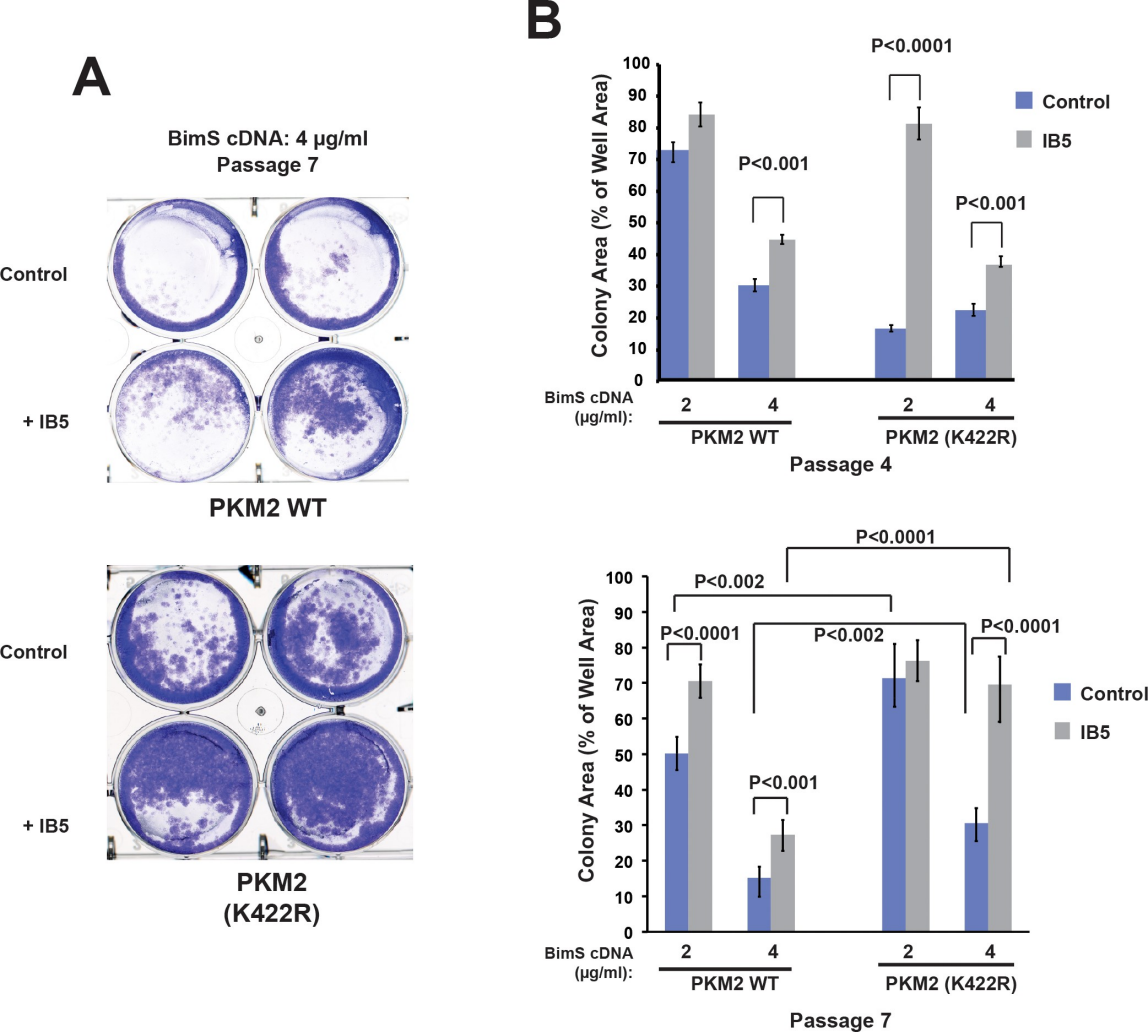
The stably tetrameric mutant PKM2 (K422R) produced cell rescue in response to expression of IB5; in long-term culture, this mutant also enhanced BimS-resistant clonogenic survival even in the absence of IB5. PKM2-deficient MEFs reconstituted with WT or mutant PKM2 cDNA were infected or not with IB5, then 1 x 10^5^ cells were plated and transfected with BimS expression plasmid. The plates were fixed and stained with crystal violet after 1 week, and the total area of colonies were counted as above. A. Example of stained colonies at passage 7. B. Quantification of clonogenic survival for passages 4 and 7. At both early and later passages following IB5 or control infection, the K422R mutant supported the cytoprotective effect of IB5; at later passage, this mutant protected cells to a substantial degree even in the absence of IB5 expression. SD and *P* values were calculated from six individual plates. Note: underlying data are included in corresponding tabs in the accompanying supplemental Excel file S1 Data. IB5, intrabody 5; MEF, Mouse Embryonic Fibroblast; PKM2, pyruvate kinase isoform M2; WT, wild-type.

In MEFs reconstituted with PKM2 variants for a short time (3–4 passages), the K422R mutant slightly increased the numbers of viable cells compared with WT PKM2, and IB5 expression further increased viability (Fig 5). At this early time, IB5 expression enhanced clonogenic survival with PKM2 (K422R) in a manner similar to WT PKM2. (For unknown reasons, survival in the absence of IB5 was somewhat reduced with this mutant, compared with WT PKM2.) However, in MEFs that had expressed the K422R mutant for a longer time (passage 7), clonogenic survival was increased even in the absence of IB5 and was further enhanced by IB5 expression (Fig 5). This argues that stably tetrameric PKM2 promoted a cell survival function that developed over time (see below).

The nuclear form of PKM2 is thought to be dimeric, whereas tetramers are restricted to the cytoplasm [54]. If so, our data imply that the anti-apoptotic effect of IB5 involves cytoplasmic PKM2 molecules and does not require the transcriptional activities ascribed to dimeric PKM2 in the nucleus. Consistent with this, we found that the K270M mutant, which formed dimers but not stable tetramers, as seen in blue native gels, even in the presence of FBP (S5A Fig) but is reportedly competent in nuclear transactivational activity [52], failed to support IB5-induced cell rescue (Fig 2A).

### A mitochondrial role in IB5-induced apoptosis resistance

Our results showed that IB5 and PKM2 inhibited apoptosis triggered by transient expression of BimS or tBid, which directly activate the “intrinsic” pathway involving Bax/Bak-dependent permeabilization of mitochondrial outer membranes. This raised the possibility that PKM2 can directly inhibit the activity or function of Bax/Bak at mitochondria. However, we observed no effect of adding recombinant scFv 5 and PKM2 to our well validated in vitro systems based on isolated mitochondria or liposomes mixed with Bax and cleaved Bid, which recapitulate the basic aspects of Bcl-2 family protein function in membranes [6,24,25,85]. Thus, we saw no evidence that PKM2 acts directly on the process of Bax/Bak-mediated MOMP.

**Fig 6:**
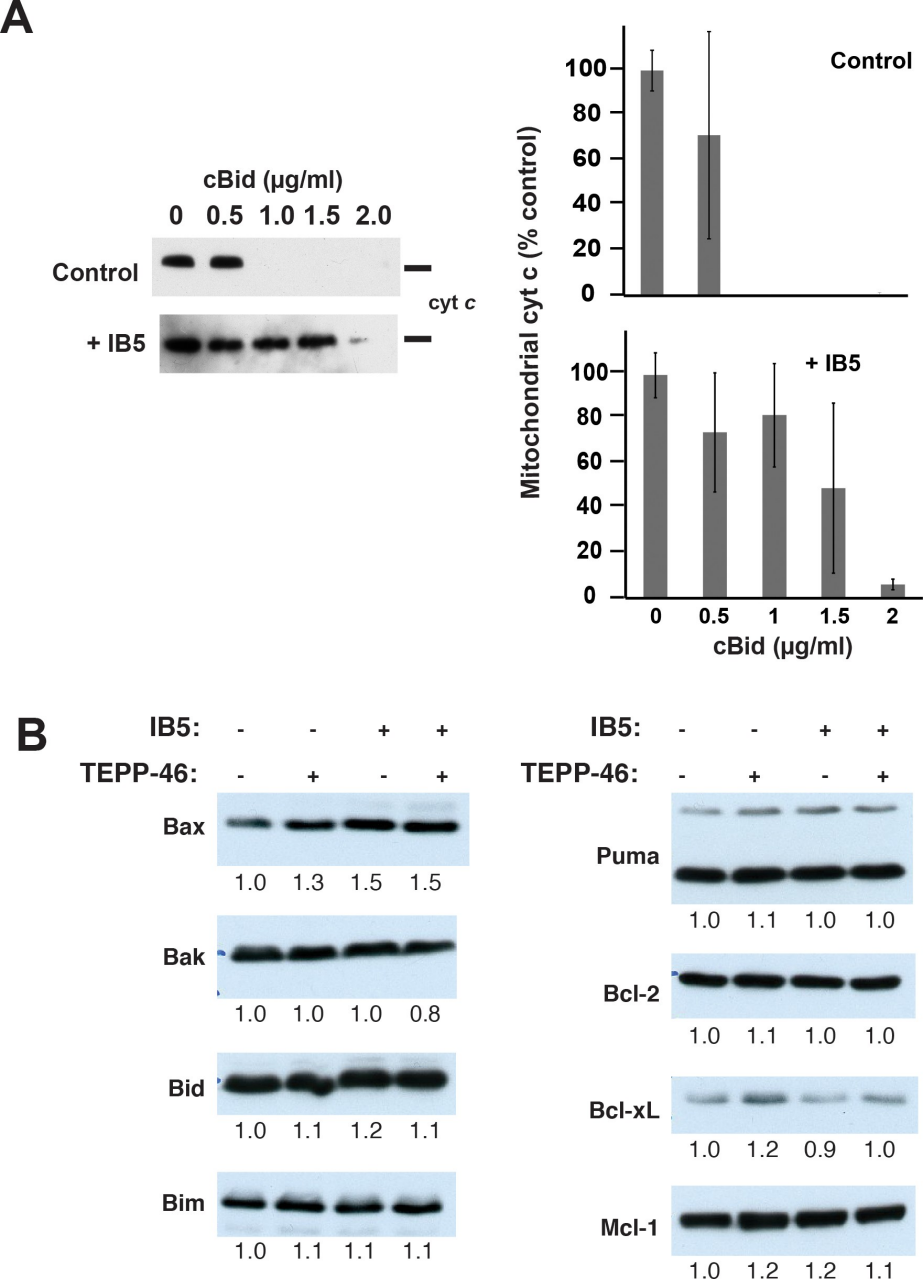
Mitochondria from cells expressing IB5 were relatively resistant to cBid-induced MOMP. A. Cyt *c* release assay. Left panel: control (top) or intrabody-expressing (bottom) 293T cells were collected, and the mitochondrial fraction was isolated by differential centrifugation. To induce MOMP, recombinant cBid protein was added at the indicated concentrations. After incubation for 30 min at 37 ºC, samples were centrifuged, and cyt *c* content in mitochondrial pellet fractions was analyzed by immunoblot. A representative of three independent experiments is shown. Right panel: densitometric quantification of average cyt *c* content ± SEM from three independent experiments. B. Levels of several Bcl-2 family proteins were unchanged following IB5 expression or incubation with TEPP-46 or both. Cell lysates from 293T cells infected with and without IB5 and incubated with and without TEPP-46 (27 μM) were separated on SDS-12% polyacrylamide gels. Bcl-2 family proteins were detected by immunoblotting. The bands were quantified using ImageJ and normalized to the control cell lysate on the leftmost lane. Note: underlying data are included in corresponding tabs in the accompanying supplemental Excel file, S1 Data. 293T, HEK293T; cBid, cleaved Bid; cyt c, cytochrome c; IB5, intrabody 5; MOMP, mitochondrial outer membrane permeabilization.

On the other hand, mitochondria isolated from 293T cells expressing IB5 were reproducibly more resistant than control mitochondria to MOMP induced by treatment with cleaved Bid protein (Fig 6A). This suggests that PKM2 could produce mitochondrial changes that could explain the cellular rescue we observed (Fig 2). As Bcl-2 family proteins are the most prominent regulators of apoptotic death at mitochondria, we first considered whether altered levels of these proteins could be responsible for MOMP resistance. In this regard, one study reported that, under conditions of oxidative stress, PKM2 can interact with and stabilize the Bcl-2 protein [86]. However, we found that IB5 expression and TEPP-46 treatment (alone or in combination) failed to change the cellular levels of Bcl-2 and other major family members Bax, Bak, Bid, Bim, Puma, Bcl-xL, and Mcl-1 (Fig 6B).

**Fig 7:**
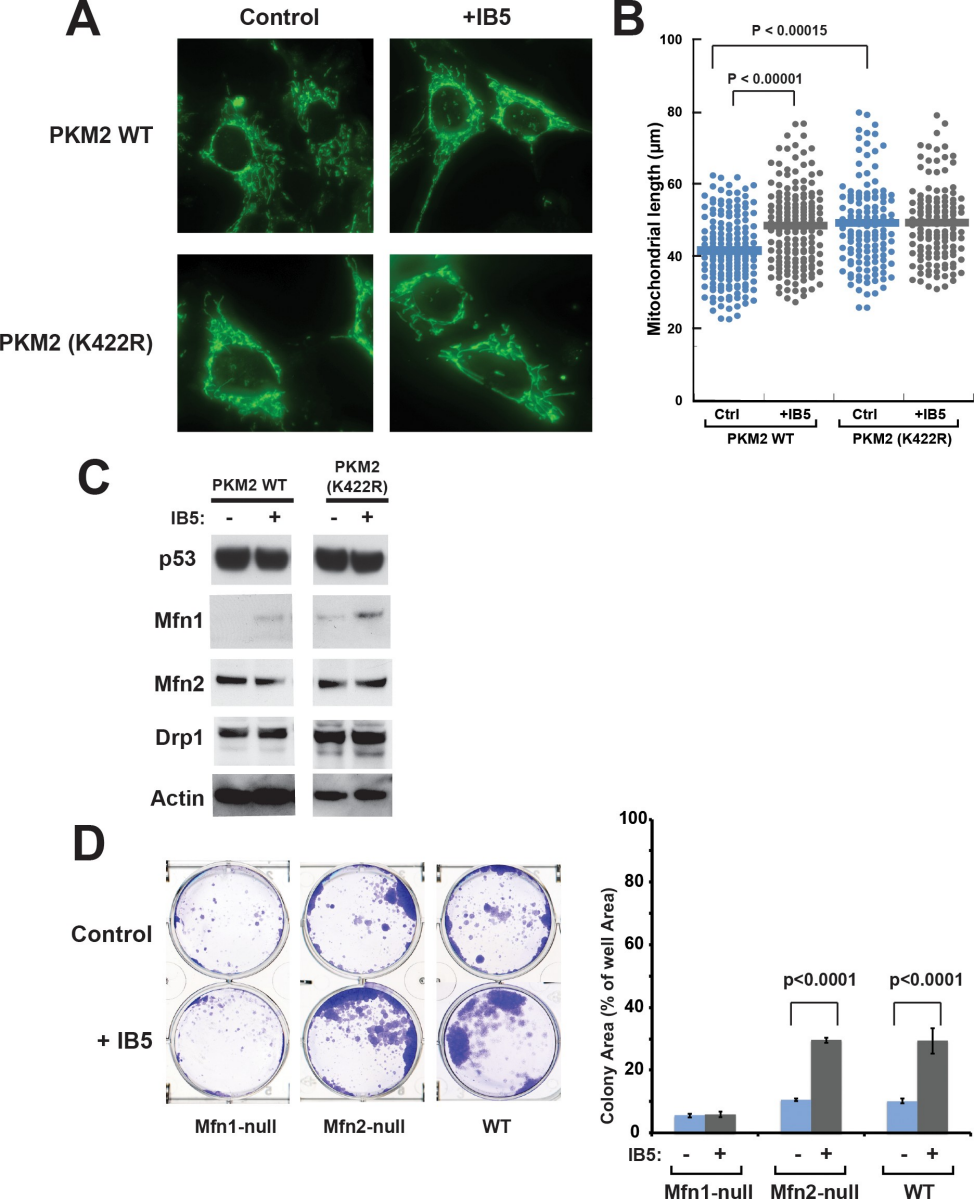
Mfn1 was required for the cytoprotective effect of IB5 and PKM2. The effect of IB5 and PKM2 on mitochondrial morphology. PKM2-deficient MEFs reconstituted with WT PKM2 or PKM2 (K422R) cDNA were infected or not with IB5 lentivirus. A. IB5 expression with WT PKM2 increased mitochondrial length, and PKM2 (K422R) expression increased mitochondrial length even in the absence of IB5. Mitochondria were visualized by fluorescence microscopy after staining with Tom20 antibodies. Representative confocal images are shown. B. Mitochondrial length scores. Cells were analyzed 3 d after transfection with the indicated cDNA constructs (mean ± SEM of 3–5 experiments of 120–200 random selected cells). C. Immunoblots showed that IB5 expression up-regulated Mfn1 protein in MEFs reconstituted with PKM2 WT and K422R mutant. IB5 expression produced no changes in the levels of Mfn2, Drp-1, or p53. K422R expression alone produced some increase in the Mfn1 level. D. IB5 expression rescued WT and Mfn2-null MEFs from BimS-induced death but failed to rescue Mfn1-null MEFs. WT, Mfn1-, or Mfn2-deficient MEFs were infected or not with IB5 lentivirus, then 1 x 10^5^ cells were plated and transfected with BimS expression plasmid. The plates were fixed and stained with crystal violet after 1 week and the total areas of colonies were measured as above. Mean, SD, and *P* values were calculated from five individual plates. Note: underlying data are included in corresponding tabs in the accompanying supplemental Excel file S1 Data. IB5, intrabody 5; MEF, Mouse Embryonic Fibroblast; Mfn, Mitofusin; PKM2, pyruvate kinase isoform M2; WT, wild-type.

We next used microscopy to analyze the effect of IB5 and PKM2 on mitochondrial morphology. We found that, in PKM2-null MEFs reconstituted with WT PKM2, IB5 expression increased the average mitochondrial length (in cells examined at passage 3–4 after transduction with IB5; Fig 7A and 7B). Furthermore, MEFs reconstituted with PKM2 (K422R) displayed a similar mitochondrial lengthening, even without IB5 expression. These results raised the possibility that PKM2-dependent mitochondrial lengthening and apoptosis resistance could involve alterations of proteins that regulate mitochondrial dynamics. In this regard, a recent study reported that PKM2 overexpression promoted mitochondrial fusion by binding to p53 and MDM2, promoting p53 ubiquitination and degradation, and thereby inhibiting expression of Drp1, a protein required for mitochondrial fission [87]. However, Fig 7C shows that the cytoprotective effect produced by IB5 was not accompanied by changes in the levels of Drp1 or p53, in MEFs reconstituted with PKM2 WT or PKM2 (K422R).

We did find that IB5 expression substantially increased the levels of Mfn1, a protein involved in mitochondrial fusion (Fig 7C), but left Mfn2 levels unchanged. Importantly, reconstituting MEFs with PKM2 (K422R) alone increased Mfn1 levels, and IB5 expression elevated Mfn1 even further (Fig 7C). To determine whether Mfn1 up-regulation occurred at the transcriptional level, we used qPCR to measure MFN1 mRNA (S6B Fig). The results show that IB5 expression did not elevate MFN1 mRNA levels but rather tended to decrease them, and IB5 + PKM2 (K422R) produced the greatest decrease. We conclude that Mfn1 protein up-regulation is post-transcriptional and can even outweigh a concomitant decrease in mRNA levels. The reason for decreased MFN1 mRNA levels is unknown, but we hypothesize that it may involve either a loss of PKM2’s nuclear functions or a gain of cytoplasmic functions, as PKM2 (K422R) is mostly tetrameric and thus expected to be excluded from the nucleus.

To determine whether Mfn1 is required for the cytoprotective effect of IB5 and PKM2, we measured BimS-resistant clonogenic survival in WT and Mfn1- or Mfn2-deficient MEFs. The Mfn1 and Mfn2 deletions were confirmed by western blot (S7 Fig). Deletion of Mfn1 or Mfn2 did not affect PKM2 expression (S7 Fig). Importantly, IB5 failed to rescue Mfn1-deficient MEFs from BimS-induced clonogenic death (Fig 7D). Even when IB5 was not expressed, Mfn1-null MEFs showed greater sensitivity to BimS-induced apoptosis than WT MEFs (Fig 7D). In contrast, Mfn2-null MEFs responded similarly to WT. These results appear consistent with a previous report that Mfn1 directly inhibits mitochondria-mediated apoptosis at the step of Bax activation, downstream of Bax mitochondrial translocation [88]. This effect did not trivially arise from an unhealthiness of Mfn1-null MEFs, as they grew at approximately the same rate as WT cells. (Note: we did not show a well untreated with BimS in Fig 7D because, in the absence of BimS transfection, both the WT and mutant cells overgrew the cultures, causing many of the cells to die and detach from the substrate.)

As mentioned above, we found that MEFs expressing PKM2 (K422R) developed a significant resistance to BimS-induced death after extended culture (passage 7), even in the absence of IB5 (Fig 5B). We hypothesize that Mfn1 up-regulation gradually increases cellular resistance to apoptosis to some extent by enhancing mitochondrial fusion. Increased fusion could be expected to gradually improve the overall health of the mitochondrial network and may, for example, limit the production of reactive oxygen species. We previously observed a similarly delayed but detrimental effect on mitochondrial function in MEFs haploinsufficient for the optic atrophy 1 (Opa1) protein [89]. In that case, inefficient mitochondrial fusion caused the late-passage cells to accumulate dysfunctional mitochondria that were deficient in Complex IV subunits.

Even in cells expressing PKM2 (K422R), which had up-regulated Mfn1 to some degree, IB5 expression further elevated Mfn1 levels and enhanced cell survival (Fig 7C and Fig 5B). Thus, the extent of survival was correlated with Mfn1 levels. Moreover, our results suggest that Mfn1 promotes survival by two different mechanisms, which could be differentially engaged depending on Mfn1 levels: 1) a slowly developing process involving enhanced mitochondrial fusion, leading to healthier mitochondria and 2) an event in which Mfn1 immediately acts to inhibit Bax/Bak-mediated MOMP [88].

How IB5 cooperates with PKM2 to up-regulate Mfn1 is unknown. One possibility is that IB5, by driving PKM2 molecules into the tetramer form, could reduce the amount of dimeric nuclear PKM2 and thereby abrogate transcriptional functions of PKM2 that could down-regulate Mfn1. Alternatively, PKM2 tetramers could act in the cytoplasm to regulate the postsynthetic degradation of Mfn1. Mfn1 turnover is reported to be controlled by E3 ligases such as MARCH5, thereby regulating apoptosis [90]. However, to our knowledge, specific proteasomal degradation of Mfn1 but not Mfn2 has not been reported.

In summary, our data show that high PK activity by itself was insufficient to produce an anti-apoptotic effect, as expression of the constitutively glycolytic PKM1 or treatment of WT cells with the PKM2-stimulator TEPP-46 did not rescue cells from BimS-induced death. This argues that the anti-apoptotic effect induced by IB5 involves a nonglycolytic activity of PKM2. On the other hand, TEPP-46 significantly enhanced the cytoprotective activity of IB5, and a stably tetrameric mutant of PKM2, K422R, enhanced the effects of IB5 and TEPP-46. Taken together, these results argue that IB5’s anti-apoptotic activity involves a tetrameric conformation of PKM2.

In the absence of IB5, cells expressing PKM2 (K422R) for multiple passages displayed a degree of apoptosis resistance, and IB5 expression further enhanced this resistance. Such a cytoprotective effect of K422R may help explain why this mutation promoted oncogenesis in mice and occurred spontaneously in Bloom syndrome patient cells [91]. Bloom syndrome involves a mutation-prone mechanism and can therefore be considered an in vivo phenotypic selection process, in effect similar to our intrabody selection approach.

Finally, the anti-apoptotic activity induced by IB5 was not accompanied by changes in the levels of major Bcl-2 family proteins. In contrast, IB5 did up-regulate Mfn1, and apoptosis resistance was ablated by Mfn1 deletion. This is consistent with reports that Mfn1 protein can oppose Bax-dependent MOMP [88]. Our observation that mitochondria isolated from IB5-expressing cells were more resistant to Bax-mediated apoptosis (Fig 6) may reflect increased levels of Mfn1 in mitochondria.

### Potential implications

PKM2-deficient cells can form tumors in mice. Often the rapidly proliferating subset of tumor cells remodel glucose utilization by expressing low PKM1 levels, whereas nonproliferating tumor cells are more likely to express higher levels of PKM1 [92]. These observations reinforce the idea that reduced PK activity, and not necessarily PKM2 expression per se, is important for rapid cell proliferation. However, they also pose a question: if PKM2 is not strictly required for tumor formation, why is PKM2 expression overwhelmingly favored in human cancers? Although some human cancers harbor PKM2 loss-of-function mutations, these mutations are typically heterozygous. Thus, cancer cells presumably benefit from retaining at least one WT allele of the M2 isoform, which, unlike M1, provides adaptive glycolytic regulation and nonglycolytic functions [38].

Our results suggest another potential benefit for cells expressing PKM2: resistance to apoptosis. Because PKM2 inhibits the central mechanism of apoptosis involving mitochondria, PKM2 could promote cell survival despite circumstances that would otherwise be cytotoxic. We can conjecture that particular subsets of neoplastic or preneoplastic cells could engage this mechanism to survive under adverse conditions, favoring oncogenesis. Because glycolytically active PKM2 typically corresponds with lower rates of proliferation, we could hypothesize that this cell survival function of active PKM2 tetramers might be seen primarily in slowly proliferating tumor cell subsets.

IB5 most likely promotes cell survival by altering the interaction of PKM2 with one or more protein partners. It is tempting to hypothesize that IB5 mimics a natural PKM2-interacting protein. However, the identity of such a putative ligand is still unknown, as are the circumstances under which it is potentially engaged. Perhaps this cell survival function of PKM2 occurs only under specific conditions (e.g., allosteric activation of PKM2 combined with another regulatory event), which may explain why it has not been identified through conventional approaches. An anti-apoptotic function of PKM2 could be important both in cancer cells and in normal cell populations that preferentially express PKM2, such as macrophages [41,93–96] and podocytes in the kidney [97,98].

## Materials and methods

### Cell culture and plasmids

Primary and immortalized WT and PKM2-deficient MEFs (PKM2^Δ/Δ^) were maintained in MEMα medium supplemented with 10% FBS, penicillin and streptomycin (Gibco-Invitrogen), and 0.1 mM of 2-mercaptoethanol [49]. 293T cells were maintained in DMEM containing 10% (vol/vol) FBS and antibiotics. pLHCX-Flag-mPKM2 (Plasmid #42512) was obtained from Addgene.

### Intrabody library construction

The intrabody scFv library was prepared using a naïve human combinatorial scFv phage library [76]. The scFv phagemid library was digested with SfiI, and the approximately 800-bp insert scFv-coding sequence was ligated into the SfiI-digested lentiviral vector, driven by an EF1α promoter (without a secretion leader sequence), followed by a FLAG tag.

### Lentiviral infection with the scFv library

Lentiviral particles were produced in 5 x 10^7^ 293T cells using pCMVD8.9 and pVSVg viral packaging vectors at a ratio of 1:1:1. For the first round of selection, culture supernatants containing lentiviral particles were collected, filtered, and used for infection of 1 × 10^7^ 293T cells per 10-mm plate. For the recloning step after rounds 2 and 3, 5 x 10^6^ cells were used. Forty-eight h post infection, the culture medium was replaced with fresh MEMα medium supplemented with 10% FBS and penicillin/streptomycin (Gibco-Invitrogen). Immunofluorescence microscopy using anti-FLAG antibody showed that over 90% of cells expressed IB5.

### Selection of intrabodies conferring BimS resistance in 293T cells

Human BimS cDNA was subcloned into pShooter mammalian expression vector (pCMV/myc/cyto; Invitrogen) to allow the expression of BimS driven by a CMV promoter. Approximately 5 x 10^7^ 293T cells were then infected with the intrabody library and then transfected with 4 μg/ml BimS plasmid using 10 μl of Lipofectamine 2000 transfection reagent (Thermo Fisher). After 24 h post infection, the culture medium was replaced with fresh MEMα medium supplemented with 10% FBS and penicillin/streptomycin (Gibco-Invitrogen).

### Recovery of selected scFv from the genomic DNA by PCR and construction of intrabody libraries for the second and third rounds of selection

The integrated intrabody-coding sequences from the surviving cells were recovered after 48 h incubation and used to construct a secondary lentiviral library, as follows. Genomic DNA from the surviving 293T cells was recovered using a DNeasy Blood & Tissue kit (Qiagen). A sample of 100 ng of the genomic DNA was used as a PCR template. A pair of primers matching the regions flanking the scFv fragment was used to amplify the integrated antibody fragment from the genomic DNA. The PCR product was digested with SfiI and inserted back into the lentiviral vector for a subsequent round of BimS selection, as described above. In total, over 300 clones with distinct DNA sequences were harvested and tested individually for the ability to confer BimS resistance. Sequences were analyzed with Vbase2.

### Expression of scFv in *E*. *coli*

scFv-coding sequences subcloned into pET28a plasmid were introduced into Rosetta (DE3)pLys cells (Novagen). Single colonies were picked and grown in 2 l of LB medium containing 50 μg/ml of kanamycin at 30°C for 8 h, then incubated for 12 h at 4°C with 0.2 mM IPTG under vigorous shaking. Cells were pelleted by centrifugation, frozen/thawed, resuspended in 50 ml of lysis buffer (Tris 25 mM pH 8.0, NaCl 300 mM), incubated 1 h on ice, and then lysed by sonication. The scFv was recovered from the soluble fraction by passage over a Ni^++^-NTA affinity column (GE Healthcare).

### Target protein immunoprecipitation

FLAG-tagged intrabody was introduced along with a tandem Strep-tag by PCR into the same lentiviral vector used for selection. 293T cells infected with the intrabody lentivirus were incubated at 30°C for 72 h, as described above. After washing with cold PBS, 5 × 10^8^ cells were lysed for 15 minutes on ice in lysis buffer (50 mM Tris HCl, pH 7.4, 150 mM NaCl, 1 mM EDTA, 1% Triton X-100). Cell lysates were clarified by centrifugation for 15 min at 4°C at 16,000 x g. The total protein content of the soluble fraction was quantified using the BCA assay. For pull-down experiments, 10 mg of protein lysate was incubated with 200 μl of EZview Red anti-FLAG M2 Affinity Gel (Sigma-Aldrich) for 2 h at 4°C. Beads were washed three times in wash buffer (50 mM Tris HCl, pH 7.4, 150 mM NaCl, 1 mM EDTA, 0.1% Triton X-100). Elution was performed under native conditions by competition with 3X FLAG peptide following the manufacturer’s protocol. Eluates were used for the second-step purification using Strep-Tactin Superflow Plus (Qiagen), following the manufacturer’s instructions. The final two-step purified protein was used for SDS-PAGE analysis. Bands of interest were cut out from the gel and subjected to in-gel digestion with trypsin (PR omega, Fitchburg, WI, USA), followed by MALDI TOF/TOF mass spectrometry analysis (Biomolecular and Proteomics Mass Spectrometry Facility, University of California at San Diego).

### Cell viability and clonogenic survival assays

Cell viability was measured with a Countess Automated Cell Counter (Invitrogen) using trypan blue. For the clonogenic survival assay, 293T or MEFs cells were seeded in 6-well plates at 1 x 10^5^ cells/well in a 2-ml volume, transfected with BimS cDNA, and incubated for 24 h. Afterwards, the medium was replaced and the cells cultured for 3–4 d. Thereafter, the plates were rinsed with PBS and fixed and stained with a solution containing crystal violet (0.5% w/v) and glutaraldehyde (6% v/v), as described [99]. The results were quantified using ImageJ software (either total cell area or number of colonies, as indicated in the figure legends).

### PKM2 protein expression and purification

pET28a-His-hPKM2 plasmid was obtained from Addgene. The PKM2 mutants pET28a-His-hPKM2 (C358S) and pET28a-His-hPKM2 (K270M) were generated by Quick-Change mutagenesis (Stratagene). All plasmids were verified by DNA sequencing and transformed into *E*. *coli* strain BL21 (DE3). WT and mutant PKM2 proteins were overexpressed in LB medium at 30°C with 200 mM IPTG for 3 h. Cells were harvested and lysed in buffer containing 25 mM Tris (pH 8.0), 300mM NaCl. The supernatants were loaded on a Ni^++^-NTA affinity column (GE Healthcare) for protein purification.

### PK assay

PK activity was measured by using Kinase-Glo Plus Luminescent Kinase Assay kit (Promega Corporation, Madison, WI, USA). Purified WT or mutant PKM2 (50–100 nM) was added in 100 μl assay buffer containing 50 mM Tris pH 7.5, 100 mM KCl, 10 mM MgCl_2_, 200 μM PEP, 200 μM ADP, and 3% DMSO. After a 15-min incubation, Kinase-Glo Plus reagent was added, according to the manufacturer’s instructions. In some cases, 0–40 μM FBP and 0–150 nM scFv 5 were added.

### Mitochondria isolation and cytochrome *c* release assay

Mitochondria were isolated from 5 x 10^8^ cells, as described [100]. The freshly isolated mitochondria (100 mg protein/ml) were then incubated with recombinant cleaved Bid protein [6] at the indicated concentrations in the presence or absence of purified scFv 5. After incubation for 30 min at 37 °C, mitochondria were collected by centrifugation at 10,000 × g and analyzed by immunoblotting, as described [100].

### Immunoblots of Bcl-2 family proteins

293T cells were washed with PBS and lysed with PBS containing 1% NP-40. Protein concentration was determined using Pierce BCA reagent (ThermoFisher; 23221, 23224). 35 μg protein was loaded in each lane of 12% SDS-PAGE gels. Proteins were transferred to nitrocellulose membrane and immunoblotted with the following primary antibodies: anti-Bax antibody (Santa Cruz N20), anti-Bak antibody (Cell Signaling 3814), anti-Bid antibody (R&D Systems AF860), anti-Bim antibody (Sigma B7929), anti-Puma antibody (Cell Signaling 4976), anti-Bcl-2 antibody (Abcam 32124), anti-Bcl-xL antibody (Cell Signaling 2764), and anti-Mcl-1 (Santa Cruz S19) at 1:1,000 dilution. The secondary anti-rabbit and mouse antibodies, conjugated with HRP, were obtained from Santa Cruz and were used at 1:2,000 dilution. The luminescence signal was detected using ECL reagent (ThermoFisher 32106).

### siRNA silencing of PKM2

Untreated or IB5-infected 293T cells (5 × 10^5^ per well) were seeded into 6-well plates (Falcon) and transfected with 30 nM siRNA. Lipofectamine 2000 (Invitrogen, Carlsbad, CA, USA) was used for transient transfection, according to the manufacturer’s protocol. After a 30-h incubation, fresh medium containing 30 nM siRNA and 4 μg/ml BimS expression plasmid was added. Cell viability assay was assayed after a further 36-h incubation. The PKM2-siRNA and control siRNA were purchased from Dharmacon (SiGENOME SMART pool hPKM2, Si156, and ON-TARGET plus nontargeting siRNA 2) [101].

### Quantification of Mfn1 mRNA expression by qPCR

Isolation of total RNA was performed using the RNeasy mini-kit (Qiagen, CA). After removal of contaminating DNA using DNase I (Invitrogen, CA), cDNA was synthesized using SuperScript III first-strand synthesis system for qPCR (Invitrogen). MFN1 mRNA levels were normalized against mouse GAPDH mRNA.

## Supporting information

S1 Fig siRNA knockdown of PKM2 ablated the protective effect of IB5 in 293T cells. Approximately 5 x 10^5^ cells were incubated per well for 12 h, then cells were either mock-transfected, transfected with 30 nM PKM2-specific siRNA (si M2), or as control, transfected with NF-κB p50-specific siRNA (si p50). After a further 36-h incubation, samples of the same siRNAs were added along with 4 μg of BimS cDNA in fresh medium. Viable cells were counted after another 48-h incubation. Note: underlying data are included in corresponding tabs in the accompanying supplemental Excel file S1 Data. 293T, HEK293T; IB5, intrabody 5; NF-κB, nuclear factor κB; PKM2, pyruvate kinase isoform M2; siRNA, small interfering RNA.
(TIF)

S2 Fig IB5 promoted clonogenic survival in cells treated with the DNA-damaging drug, etoposide. Approximately 5 x 10^5^ HCT116 and U2OS cells were plated and transfected with BimS expression plasmid including 150 nM etoposide or 1 μM Staurosporine. The plates were fixed and stained with crystal violet after 5 d and the total areas of colonies were measured. Mean, SD, and *P* values were calculated from three individual plates. Note: underlying data are included in corresponding tabs in the accompanying supplemental Excel file S1 Data. IB5, intrabody 5
(TIF)

S3 Fig IB5 failed to rescue breast cancer–derived cell lines MDA-MB231 and lung metastatic derivative MDA-MB231-LM2 from BimS-induced cell death. Control or IB5-expressing cells were transfected with BimS cDNA. The plates were fixed and stained with crystal violet after 12 days and the total areas of colonies were measured. Mean, SD, and *P* values were calculated from three individual plates. Note: underlying data are included in corresponding tabs in the accompanying supplemental Excel file S1 Data. BimS, short isoform of BimS; IB5, intrabody 5
(TIF)

S4 Fig Expression of IB5 had no effect on expression of endogenous PKM2 or Bim EL and L isoforms. 293T cells were infected (lane 2, 3) or not (lane 1) with IB5 lentivirus and incubated with (lane 3) or without 2 μg of BimS cDNA (lane1, 2) in fresh medium. Cells were lysed, and total cell protein extracts were subjected to western blot analysis. BimEL (upper band), BimL (middle band) and BimS (lower band) were detected using Anti-Bim antibody (ab15184). GAPDH was used as loading control. 293T, HEK293T; GAPDH, glyceraldehyde phosphate dehydrogenase; IB5, intrabody 5; PKM2, pyruvate kinase isoform M2
(TIF)

S5 Fig The glycolysis-defective mutant PKM2 (K367M) failed to support cell rescue in response to IB5 expression, but also formed a species with aberrant electrophoretic mobility. A. PKM2-deficient MEFs reconstituted with WT or mutant PKM2 cDNA were infected or not with IB5, then 2 x 10^4^ cells were plated and transfected with BimS expression plasmid. The plates were fixed and stained with crystal violet after 1 week and the total area of colonies were counted as above. Means, SDs, and *P* values were calculated from three experiments. B. Blue native gel electrophoresis of PKM2 WT and mutations. C. scFv 5 stimulated glycolytic activity of WT PKM2 and PKM2 (K367M). Activity was measured as in Fig 4. Note: underlying data are included in corresponding tabs in the accompanying supplemental Excel file S1 Data. IB5, intrabody 5; MEF, Mouse Embryonic Fibroblast; PKM2, pyruvate kinase isoform M2; scFv, single-chain variable fragment; WT, wild-type
(TIF)

S6 Fig Aspects of the mechanism of IB5 action. A. 2-deoxy-D-glucose had no effect on 293T cell survival induced by IB5 intrabody. 293T cells were infected or not with IB5, then 2 x 10^4^ cells were plated and transfected with BimS expression plasmid. The glycolytic inhibitor 2-deoxy-D-glucose (20 mM) was added to the MEMαmedium, and after 24 h, cells were transfected or not with 1 μg of BimS cDNA in fresh medium. The plates were fixed and stained with crystal violet after 1 week. B. IB5 reduced MFN1 mRNA levels, implying that Mfn1 protein up-regulation is post-transcriptional. PKM2-deficient MEFs reconstituted with WT or mutant PKM2 cDNA were infected or not with IB5, and MFN1 mRNA levels were quantified by qPCR. Means, SDs, and *P* values based on four independent experiments are indicated. Note: underlying data are included in corresponding tabs in the accompanying supplemental Excel file S1 Data. 293T, HEK293T; IB5, intrabody 5; MEMα; PKM2, pyruvate kinase isoform M2; WT, wild-type
(TIF)

S7 Fig Confirmation of MFN1/2 deletion in MEFs and a lack of effect on PKM2 levels. Lysates from the indicated MEF strains were analyzed by immunoblotting with antibodies directed against Mfn1, Mfn2, and PKM2, as indicated. Actin and GAPDH were used as loading controls. GAPDH, glyceraldehyde phosphate dehydrogenase; MEF, Mouse Embryonic Fibroblast; Mfn, Mitofusin; PKM2, pyruvate kinase isoform M2
(TIF)

S1 Data Data underlying figures and supporting information figures. (XLSX)

## Abbreviations

293T: HEK293T; BH3, Bcl-2 homology 3 domain
EGFR: epidermal growth factor receptor
FBP: fructose-1,6-bisphosphate
IB5: intrabody 5
LPS: lipopolysaccharide
MEF: Mouse Embryonic Fibroblast
MALDI-TOF: matrix-assisted laser desorption/ionization-time-of-flight
Mfn1: Mitofusin-1
MOM: mitochondrial outer membrane
MOMP: mitochondrial outer membrane permeabilization
PEP: phosphoenolpyruvate
PKM1/M2: pyruvate kinase isoform M1/M2
scFv: single-chain variable fragment
siRNA: small interfering RNA
T3: triiodo-L-thyronine
V_H_: immunoglobulin heavy chain
V_L_: immunoglobulin light chain
WT: wild-type
