# Supplementary figures and images for: Phenotypic selection with an intrabody library reveals an anti-apoptotic function of PKM2 requiring Mitofusin-1
Source: PLoS Biol. 2019 Jun 10;17(6):e2004413. doi: 10.1371/journal.pbio.2004413 (PMC6586363; doi:10.1371/journal.pbio.2004413)

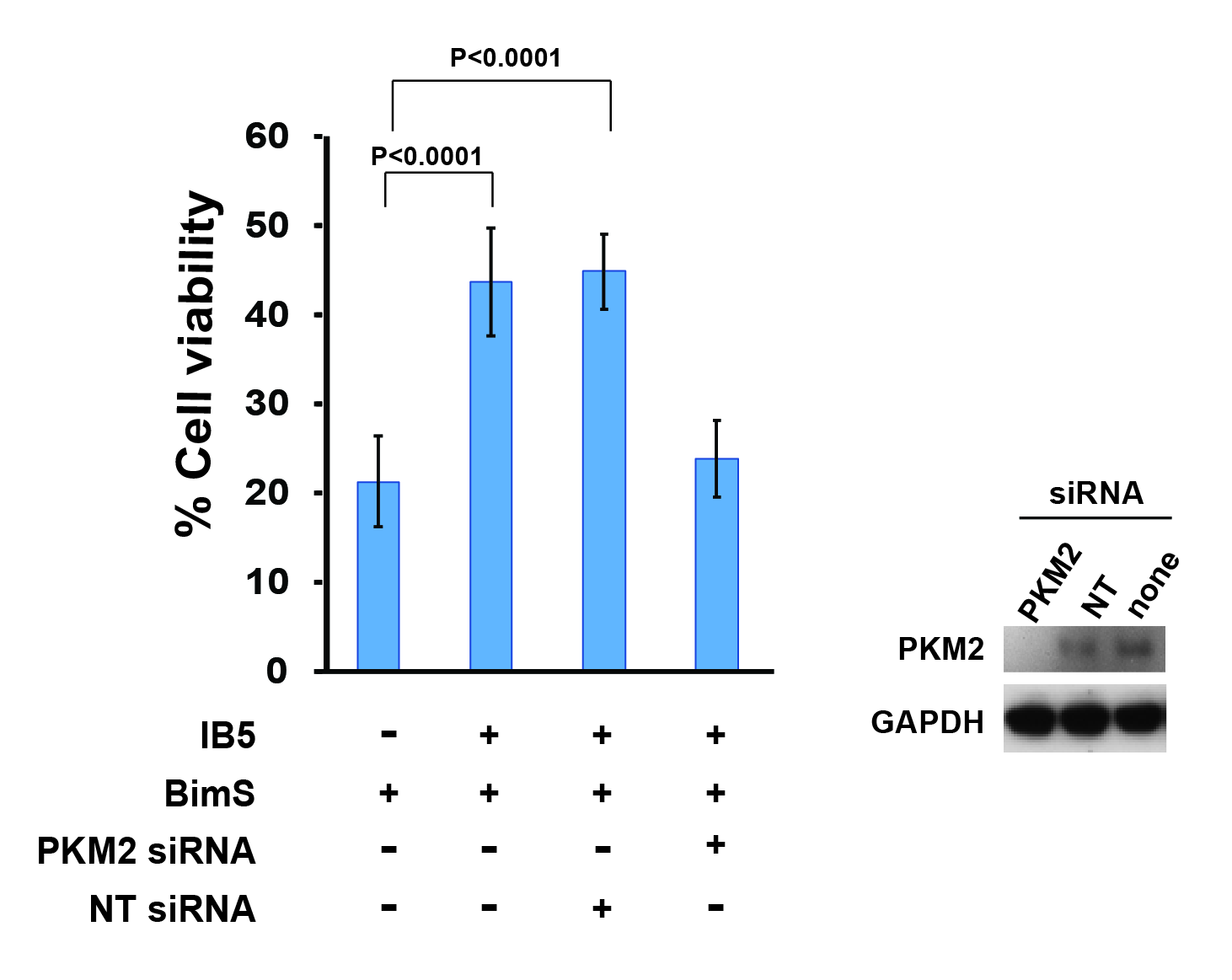

Supplement: S1 Fig — Approximately 5 x 10^5^ cells were incubated per well for 12 h, then cells were either mock-transfected, transfected with 30 nM PKM2-specific siRNA (si M2), or as control, transfected with NF-κB p50-specific siRNA (si p50). After a further 36-h incubation, samples of the same siRNAs were added along with 4 μg of BimS cDNA in fresh medium. Viable cells were counted after another 48-h incubation. Note: underlying data are included in corresponding tabs in the accompanying supplemental Excel file S1 Data. 293T, HEK293T; IB5, intrabody 5; NF-κB, nuclear factor κB; PKM2, pyruvate kinase isoform M2; siRNA, small interfering RNA. (TIF) [file pbio.2004413.s001.tif]

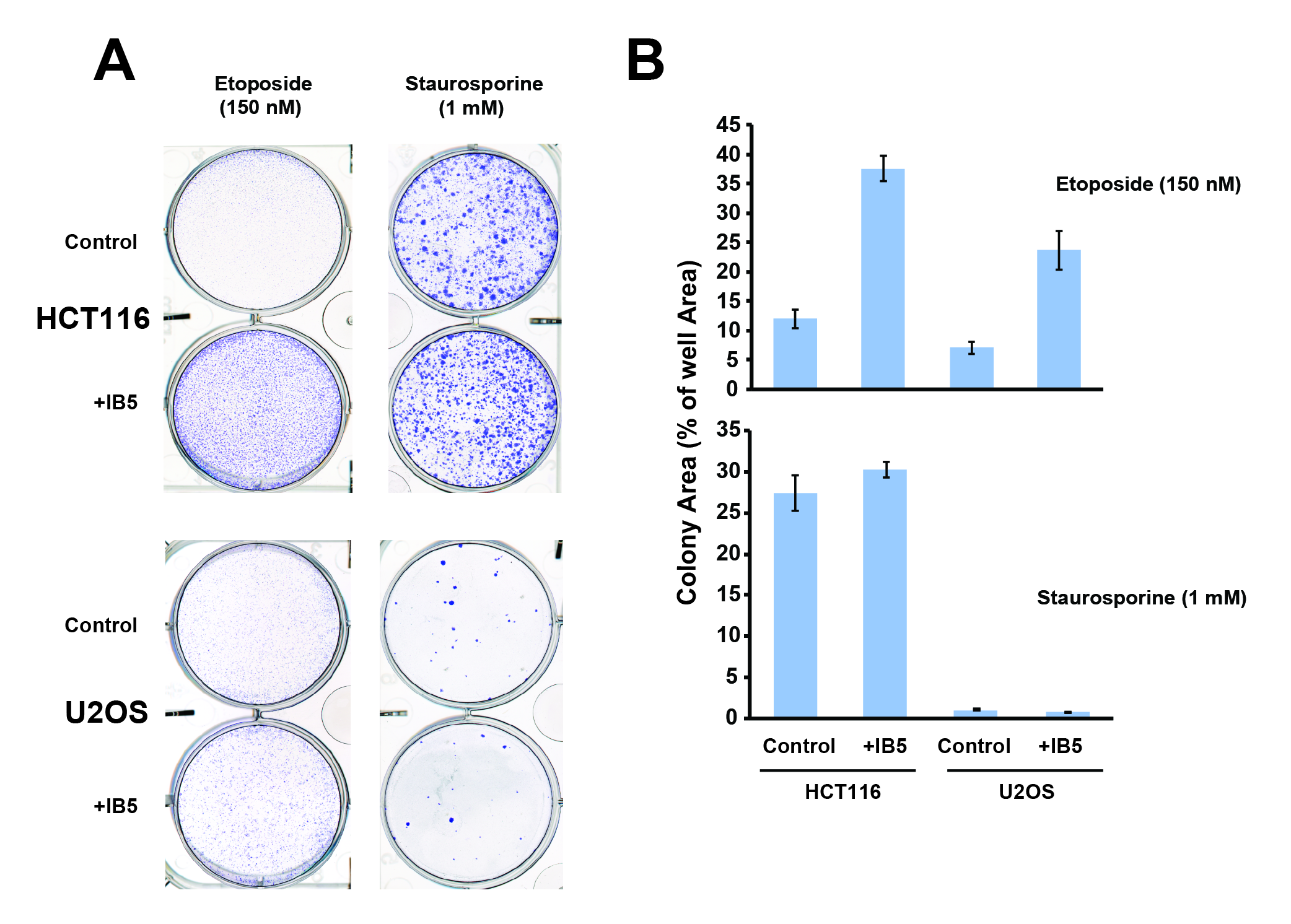

Supplement: S2 Fig — Approximately 5 x 10^5^ HCT116 and U2OS cells were plated and transfected with BimS expression plasmid including 150 nM etoposide or 1 μM Staurosporine. The plates were fixed and stained with crystal violet after 5 d and the total areas of colonies were measured. Mean, SD, and P values were calculated from three individual plates. Note: underlying data are included in corresponding tabs in the accompanying supplemental Excel file S1 Data. IB5, intrabody 5 (TIF) [file pbio.2004413.s002.tif]

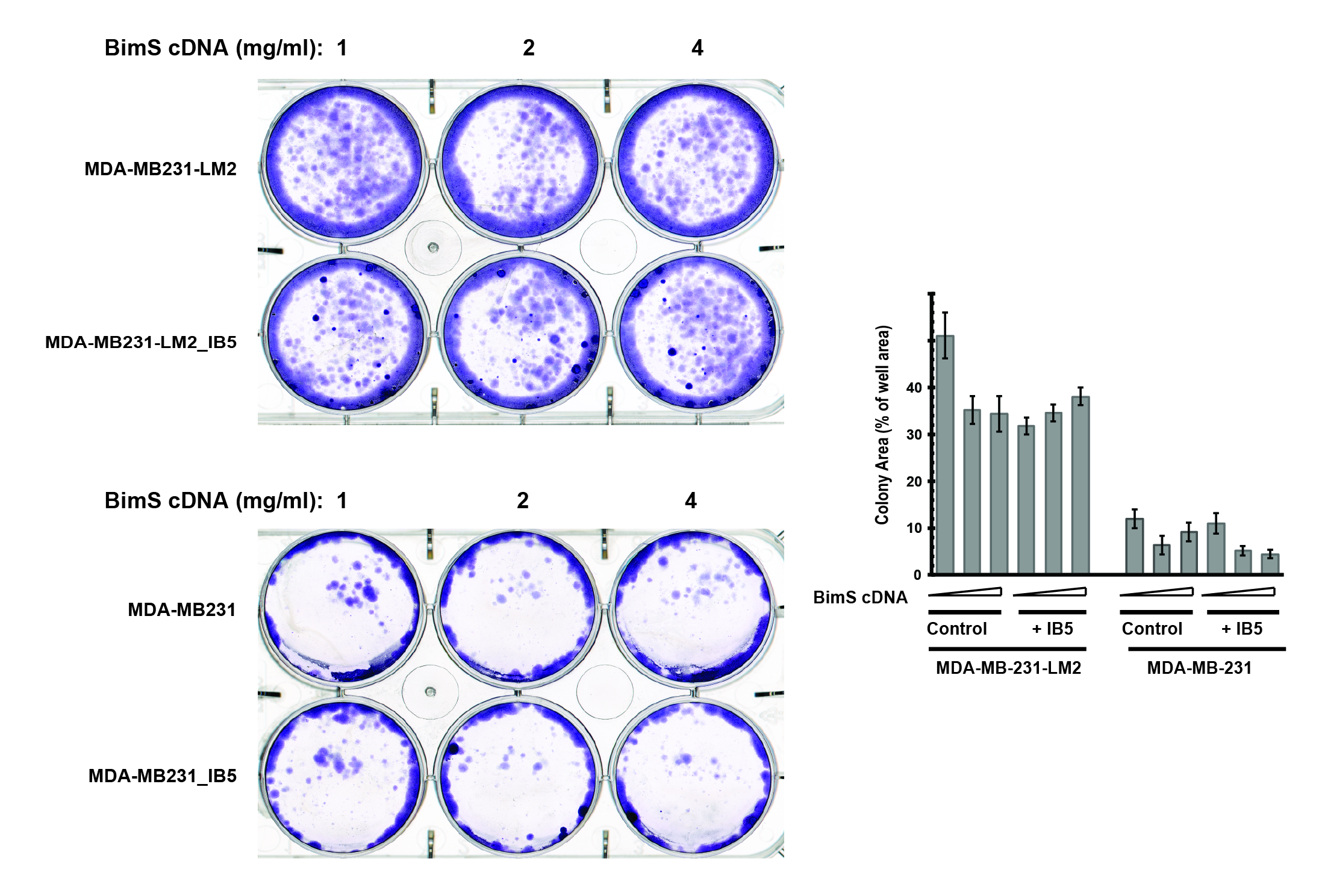

Supplement: S3 Fig — Control or IB5-expressing cells were transfected with BimS cDNA. The plates were fixed and stained with crystal violet after 12 days and the total areas of colonies were measured. Mean, SD, and P values were calculated from three individual plates. Note: underlying data are included in corresponding tabs in the accompanying supplemental Excel file S1 Data. BimS, short isoform of BimS; IB5, intrabody 5 (TIF) [file pbio.2004413.s003.tif]

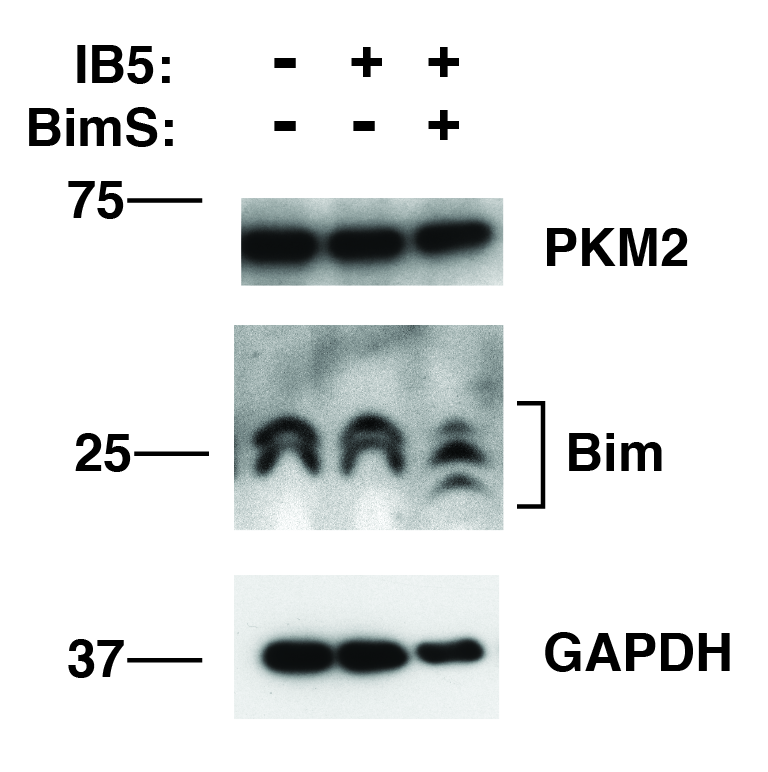

Supplement: S4 Fig — 293T cells were infected (lane 2, 3) or not (lane 1) with IB5 lentivirus and incubated with (lane 3) or without 2 μg of BimS cDNA (lane1, 2) in fresh medium. Cells were lysed, and total cell protein extracts were subjected to western blot analysis. BimEL (upper band), BimL (middle band) and BimS (lower band) were detected using Anti-Bim antibody (ab15184). GAPDH was used as loading control. 293T, HEK293T; GAPDH, glyceraldehyde phosphate dehydrogenase; IB5, intrabody 5; PKM2, pyruvate kinase isoform M2 (TIF) [file pbio.2004413.s004.tif]

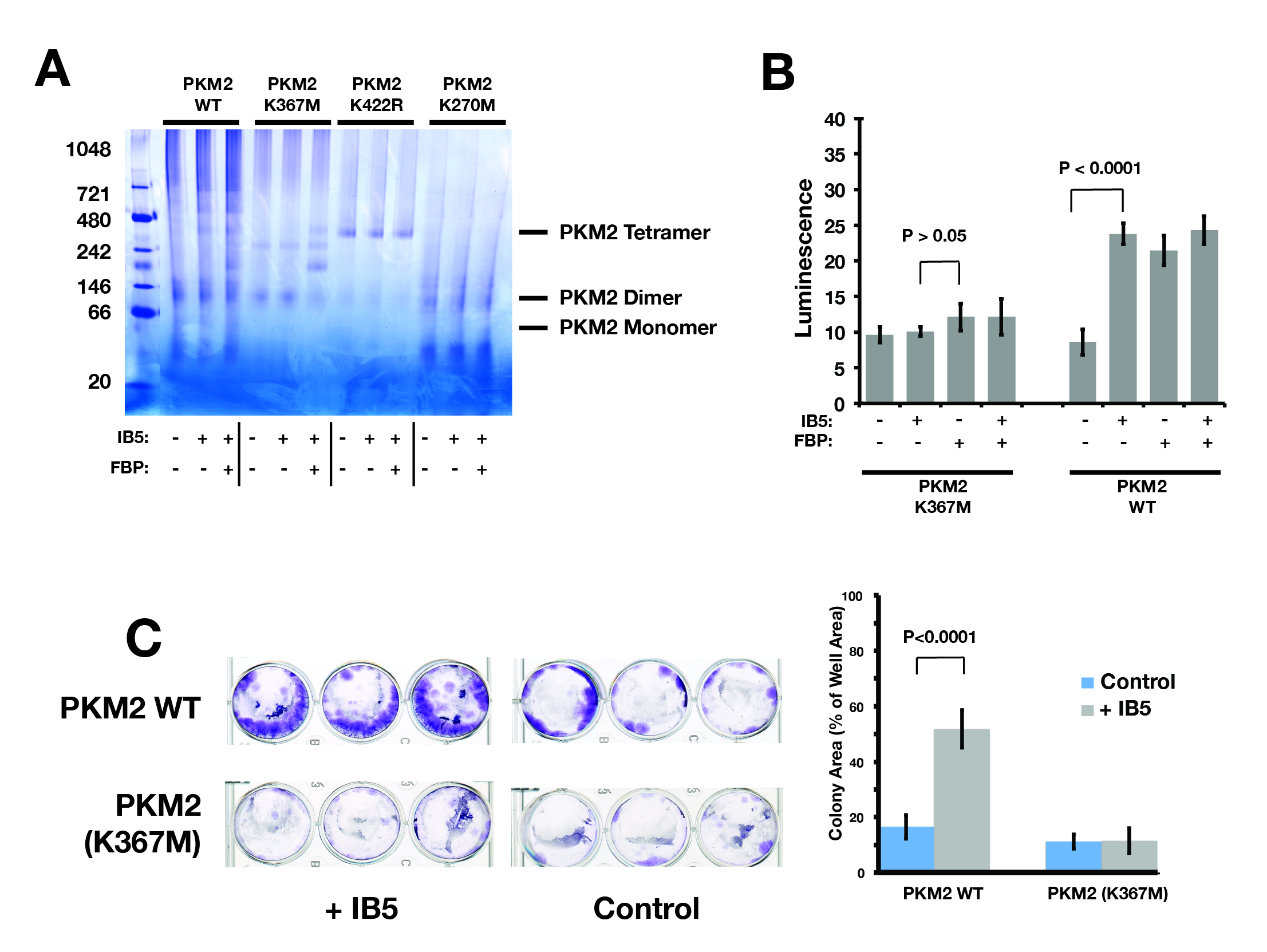

Supplement: S5 Fig — A. PKM2-deficient MEFs reconstituted with WT or mutant PKM2 cDNA were infected or not with IB5, then 2 x 10^4^ cells were plated and transfected with BimS expression plasmid. The plates were fixed and stained with crystal violet after 1 week and the total area of colonies were counted as above. Means, SDs, and P values were calculated from three experiments. B. Blue native gel electrophoresis of PKM2 WT and mutations. C. scFv 5 stimulated glycolytic activity of WT PKM2 and PKM2 (K367M). Activity was measured as in Fig 4. Note: underlying data are included in corresponding tabs in the accompanying supplemental Excel file S1 Data. IB5, intrabody 5; MEF, Mouse Embryonic Fibroblast; PKM2, pyruvate kinase isoform M2; scFv, single-chain variable fragment; WT, wild-type (TIF) [file pbio.2004413.s005.tif]

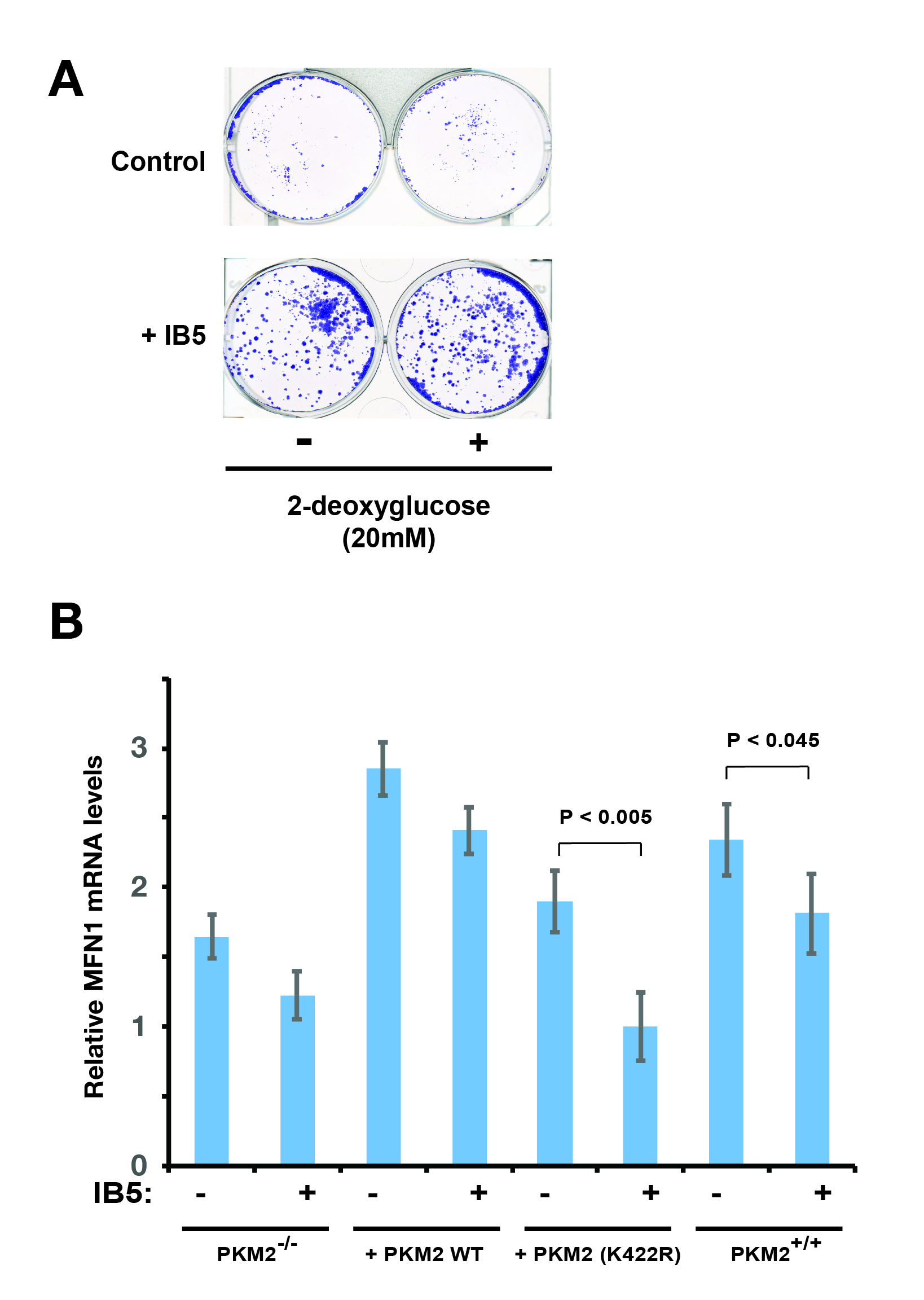

Supplement: S6 Fig — A. 2-deoxy-D-glucose had no effect on 293T cell survival induced by IB5 intrabody. 293T cells were infected or not with IB5, then 2 x 10^4^ cells were plated and transfected with BimS expression plasmid. The glycolytic inhibitor 2-deoxy-D-glucose (20 mM) was added to the MEMαmedium, and after 24 h, cells were transfected or not with 1 μg of BimS cDNA in fresh medium. The plates were fixed and stained with crystal violet after 1 week. B. IB5 reduced MFN1 mRNA levels, implying that Mfn1 protein up-regulation is post-transcriptional. PKM2-deficient MEFs reconstituted with WT or mutant PKM2 cDNA were infected or not with IB5, and MFN1 mRNA levels were quantified by qPCR. Means, SDs, and P values based on four independent experiments are indicated. Note: underlying data are included in corresponding tabs in the accompanying supplemental Excel file S1 Data. 293T, HEK293T; IB5, intrabody 5; MEMα; PKM2, pyruvate kinase isoform M2; WT, wild-type (TIF) [file pbio.2004413.s006.tif]

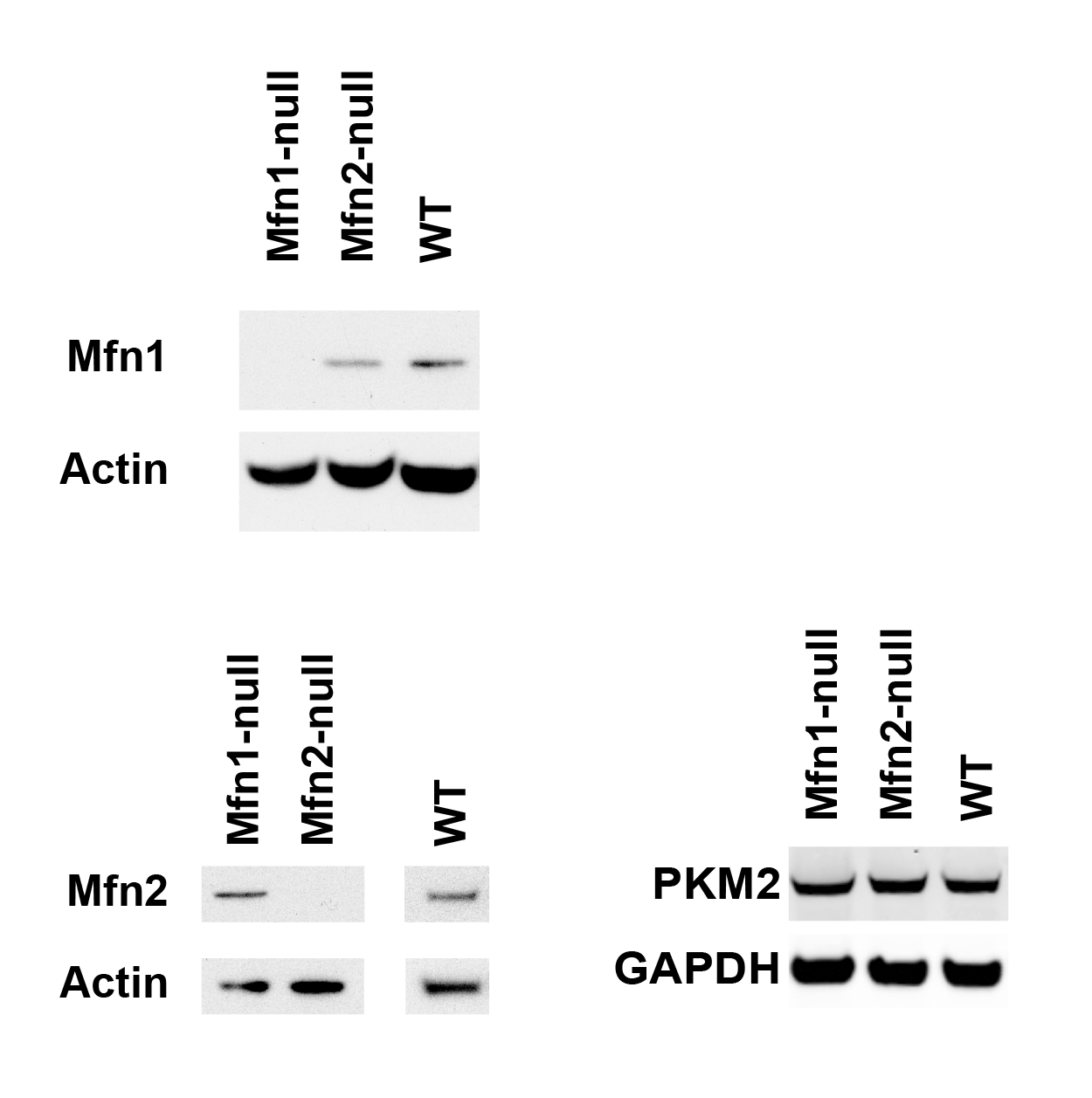

Supplement: S7 Fig — Lysates from the indicated MEF strains were analyzed by immunoblotting with antibodies directed against Mfn1, Mfn2, and PKM2, as indicated. Actin and GAPDH were used as loading controls. GAPDH, glyceraldehyde phosphate dehydrogenase; MEF, Mouse Embryonic Fibroblast; Mfn, Mitofusin; PKM2, pyruvate kinase isoform M2 (TIF) [file pbio.2004413.s007.tif]
